# Differential allocation of parental investment and the trade-off between size and number of offspring

**DOI:** 10.1098/rspb.2018.1074

**Published:** 2018-08-01

**Authors:** Irja Ida Ratikainen, Thomas Ray Haaland, Jonathan Wright

**Affiliations:** Department of Biology, Centre for Biodiversity Dynamics, Norwegian University of Science and Technology (NTNU), Høgskoleringen 5, 7491 Trondheim, Norway

**Keywords:** reproductive compensation, sexual selection, parental effects, parental care adjustment, state-dependent model, quality–quantity trade-off

## Abstract

When parents decide how much to invest in current versus future offspring and how many offspring to divide their current investments between, the optimal decision can be affected by the quality of their partner. This differential allocation (DA) is highly dependent on exactly how partner quality affects reproductive costs and offspring benefits. We present a stochastic dynamic model of DA in which females care for a series of clutches when mated with males of different quality. In each reproductive event, females choose the size and number of offspring. We find that if partner quality affects reproductive costs, then DA in total reproductive investment occurs only via changes in the number of offspring. DA in the optimal size of the offspring occurs only if partner quality affects the offspring benefit function. This is mostly in the form of greater female investment per offspring as male quality decreases. Simultaneously, we find that adaptive DA increases the number of offspring, and thus the amount of total investment, as male quality increases. Only certain model scenarios produce the positive DA in offspring size seen in empirical studies, providing a predictive framework for DA and how partner quality affects reproductive costs and offspring benefits.

## 1. Introduction

Parents face a series of critical decisions regarding reproduction [1–3], which incurs costs both in males and females [4,5]. The first major decision is whether to reproduce at all, and if so then how much to invest in any current offspring. The optimal choice maximizes lifetime reproductive success by trading off current versus future reproduction. Such decisions are affected by many factors, including how the costs of reproduction tracks increases in current investments, plus factors that may affect the marginal returns on parental investments in current versus expected future offspring. Many features of the environment may affect these trade-offs, but parental quality is often an important one [6]. Therefore, the quality of one's partner can have a strong effect on the value of current offspring [7–9]. ‘Differential allocation’ (DA) describes adjustments a parent makes to investment in offspring produced with different partners [10,11]. Positive DA describes increasing investment with partner quality, while negative DA involves greater investment in offspring from lower-quality partners [7], a pattern also termed reproductive compensation [12–14].

In multiple-offspring broods, identifying positive and negative DA is not as straightforward as observing a change in a single component of parental investment when mate quality changes. Parental investment is a composite trait, consisting of a number of different elements. Commonly used response variables in DA studies range from egg size [15–20], clutch size [21–24] and egg contents such as proteins, hormones and carotenoids [18,19,25–28] to parental feeding rates [11,29–33]. Less common traits used in this context include probability of breeding [34], date of laying onset [24], number of broods per season [35], intra-clutch variation in offspring and offspring sex or sex ratios [36,37]. Whether each trait is suitable to determine the presence or absence of DA obviously depends on the taxon in question and the presence of correlated responses in other parental investment and life-history traits. However, without carefully considering the underlying theory, especially regarding the multiple effects of mate quality on reproductive benefits and costs [38,39], any result, or lack thereof, in any given trait could be misinterpreted when considered in isolation. As an example of this challenge, Galeotti *et al*. [40] showed that female freshwater crayfish (*Austropotamobius pallipes*) laid larger eggs but smaller clutches when mated with small males with relatively larger chelae, but they laid smaller eggs and larger clutches for large males with relatively smaller chelae. In order to interpret results such as these in an adaptive perspective, we need to understand how the optimal offspring size versus number trade-off differs for females under specific scenarios of male quality effects.

Depreciable parental investment (*sensu* [3]) is investment that parents must divide among multiple current offspring, such as the energetic contents going into an egg, whereas predator defence, for example, represents a form of non-depreciable parental care, affecting all offspring equally independently of the number of offspring. This points to another and much more well understood trade-off that parents face: the one between number and quality or size of offspring [41]. If a parent is free to divide its resources among as many offspring as it likes, it should do so based upon the marginal gain of increasing investment in the quality or size of each individual offspring. In other words, the parent should maximize the units of offspring fitness it will gain for each unit of investment it makes, and this takes into account that it is the surviving number of reproductive offspring that matters for fitness rather than just the number of offspring produced. Interestingly for DA theory, this may depend upon the quality of the partner [39].

All the components of these trade-offs in parental care have been investigated in empirical studies of DA (see [7,8,39]). However, the lack of a comprehensive predictive theoretical framework for DA when parental care is depreciable has resulted in a less than systematic empirical approach. Here, we present a stochastic dynamic optimization model that investigates some potentially adaptive explanations for the DA patterns observed in nature. Specifically, we model how partner quality affects how the parent adjusts the total level of investment and the allocation of investment among different offspring. Throughout, we discuss how females adjust their investments according to the quality of the father of their offspring, but the exact same logic would hold for a male adjusting his paternal investment according to the quality of the mother of the offspring. We investigate how optimal investment in offspring depends on partner quality effects via the elevation or the slope of (i) the offspring fitness function and/or (ii) the parental cost function of the mother. When partner quality effects are multiplicative (affecting the slope of either of the functions), a single-offspring (i.e. non-depreciable care) model predicts strong DA patterns, but when male quality effects are additive (i.e. affecting only the elevation), no DA in (i) and very weak DA in (ii) is predicted [38]. These simple predictions may hold in the cases when parental care is non-depreciable, but they may change when adding the possibility of producing several offspring and thus the trade-off between offspring quality and quantity. We therefore extend the model presented by Haaland *et al*. [38] and investigate different basic scenarios, varying both offspring fitness functions and maternal cost functions systematically. We then explore some more complex, but possibly more biologically realistic, scenarios. Our model allows us to produce a framework for understanding the effects of DA on investments per offspring versus offspring number in order to provide more complete predictions for future empirical studies.

## 2. Model description

The model core is the same as the stochastic dynamic model in [38], although the notation has been changed to facilitate the extension that allows for multiple offspring. This model follows females throughout their lives as they make a series of decisions concerning their investment in current reproduction (*u*) each time step, but here females also decide how many offspring to divide their resources between (*o*). Female energetic state, *X*, is tracked throughout life, and decisions are state-dependent based upon energetic reserves, age and the quality of the male paired to in the current time step. Maximum energetic reserves are *x*_max_ = 100 and the lower critical level of reserves is *x*_crit_ = 2, below which females die. There are three classes of males in the model (low, medium and high), and the distribution of males between these classes stays constant throughout one model run. The baseline distribution of males is ***P*** = {0.3, 0.4, 0.3} of low, medium and high quality, respectively. The female is paired to a random male from this distribution each reproductive event or time step. The survival probability until next breeding season, *α*, is constant over time and low enough (*α* = 0.8 unless otherwise stated) that no females survive until the end (100 time steps). Therefore, our results are not complicated by any effects of adaptive terminal investment. Each time step, the female has a given probability, *λ*, of finding food that will add to her energetic state, and the value of food, *y*, is set to 30 in all model runs.

For each combination of state variables (age, energetic state, partner quality), the optimal investment is determined. The optimal investment consists of the combination of total investment, u*(*x*, *m*, *t*), and the number of offspring o*(*x*, *m*, *t*) that give the highest expected lifetime reproductive success. Reproductive success in the current breeding attempt is given by the multiplication of the number of offspring, *o*, with the offspring fitness given by the offspring fitness function, dependent on per-offspring investment, *u/o*. This offspring fitness function is specific to the male that the female is paired with, and dependent upon the level of female reproductive investment, *b_m_*(*u*/*o*). Female reproductive investment has a cost, manifested as a reduction in her energetic reserves, and this cost can also be male-specific, *c_m_*(*u*). The fitness obtained for a female in state *x*, with male of quality *m*, at time *t*, given an investment of *u* divided between *o* offspring, will then be2.1
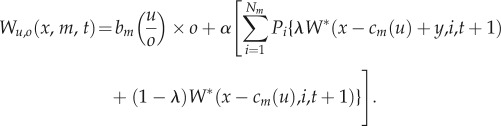


Fitness achieved by employing the optimal strategy, W*, is found by using the dynamic programming equation [42,43],2.2
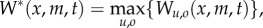
and the combination of *u* and *o* producing the highest *W_u,o_* is saved as u*(*x, m, t*) and o*(*x, m, t*).

Offspring fitness is given by2.3
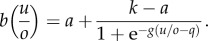


Note that the scale of fitness is completely arbitrary, and any fitness value is only meaningful in comparison to other values, given the chosen parameters.

Cost of reproduction is given by2.4
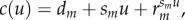
where *a*, *k*, *g*, *q*, *d*, *r* and *s* are parameters describing the exact shape of the offspring fitness—lower asymptote (baseline: −11), upper asymptote (baseline: 11), growth rate (baseline: 0.2) and inflection point (baseline: 5)—and cost functions—intercept (baseline: 0), shape (baseline: 1.3) and slope (baseline: 0.2) (see also electronic supplementary material, table S1).

We also investigated the possible effects of changing other parameters (mortality rate, environmental stochasticity, distribution of males) in the model. None of these parameter changes had any qualitative effects on the results, and only produced minor quantitative effects except for mortality rate that has a relatively strong effect of reducing any patterns in DA when high (results not shown).

We use our results to simulate what a range of individual female reproductive patterns of investment would look like in populations where these optimal decisions are being applied. To verify that DA patterns found by the stochastic dynamic programme can actually be expected to be seen in a real population, we simulated lifetime trajectories of individual females by running forward simulations. These simulations used exactly the same model parameters as the scenario under investigation and females in the simulations invested in both number and total investments according to the optimal investments from the model output.

The entire model was created in R v. 3.3.1 [44], and the code uses the additional package abind [45].

## 3. Scenarios and results

**Figure 1.**
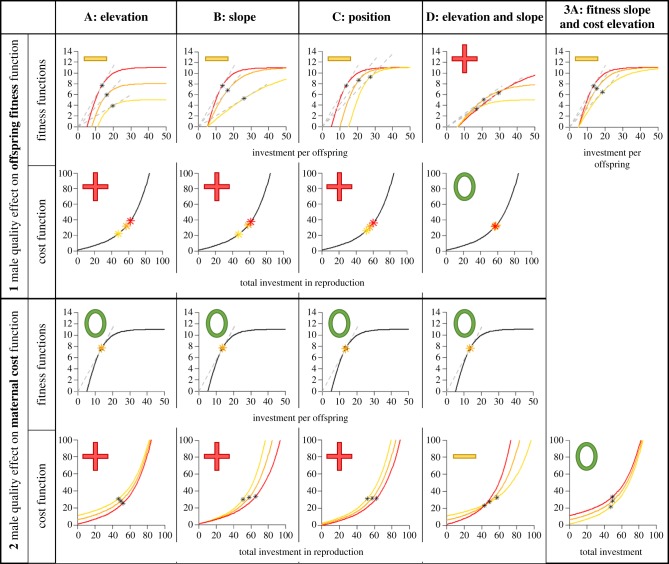
Overview of all basic scenarios presented. Upper rows (row 1 and row 3) of plots show offspring fitness functions in the different scenarios, and lower rows (row 2 and row 4) shows cost functions in the same scenarios. Note that the cost function is the same in all scenarios when the male affects the offspring fitness function, and likewise the offspring fitness function is the same in all scenarios when the male affects the maternal cost function. The red (darker) lines always represent the high-quality male, while the orange and yellow (lighter) lines represent the medium- and low-quality males, respectively. The grey dashed lines in the fitness functions represent the tangent from the origin to each of the fitness functions, and in all cases represented in this figure, they hit the fitness function in the same place as where the optimal per-offspring investment is found (represented by a star). The red ‘+’, yellow ‘–’ and green ‘0’ signs placed in each panel indicate whether the predicted DA is positive, negative or not present (colours correspond to the male quality receiving the highest investment). In the top row, it is the investment per offspring DA (i.e. offspring size) that is represented, while in the bottom row, these signs indicate DA in total investment. This can also be seen by comparing where on the *x*-axis the stars indicating the optimal investments for each male quality falls, and when the red star is left of the yellow star investments will be higher for low-quality males. (Online version in colour.)

All results are reported in detail in electronic supplementary material, S1 and summed up in figure 1. Here, we describe all scenarios, but report only the main findings and highlight the results we consider most illuminating. Each scenario shows different ways that male quality may affect the fitness consequences of female reproductive investment decisions, and we divide these scenarios into two classes in the same way as Haaland *et al*. [38]. The first class of scenarios (1A–D) involves male quality effects on the elevation and the slope of the offspring fitness function. We also explore the possibility of male quality affecting the position of the offspring fitness function along the *x*-axis (parental investment). After first investigating each of these conceptually distinct possibilities in turn, we move on to some more complex combinations that they may be more realistic in many cases.

The second class of scenarios (2A–D) involves male effects on maternal costs. For these, we also go systematically through the basic scenarios where male quality affects elevation, slope and position of the female cost function, followed by some examples of combinations of these and other scenarios where offspring number is constrained. We end by investigating one scenario (3A) where the quality of the male affects both offspring fitness function and maternal reproductive cost function. All scenarios and results are summarized in figure 1.

### (a) Male quality effects on the offspring fitness function

In these scenarios, males of different qualities affect the fitness function of each individual offspring. Such effects could be genetic or environmental through a wide range of paternal effects such as provisioning or the habitat quality available to the offspring [38].

#### (i) Male quality effects on the elevation of the offspring fitness function: scenario 1A

**Figure 2.**
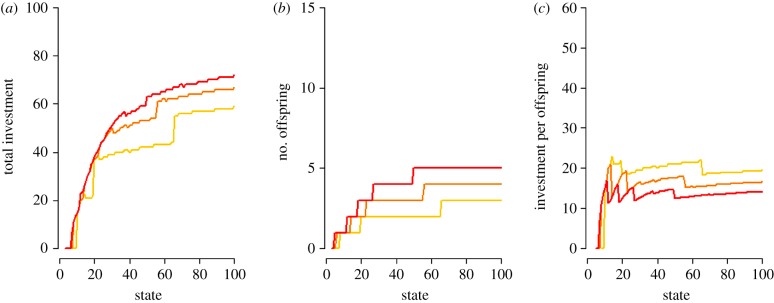
Scenario 1A (figure 1). Plots showing results from the stochastic dynamic optimization. In each plot, the different optimal investments along the axis of female energetic state (*x*-axis). Yellow lines correspond to low-quality males, orange medium-quality and red high-quality males. (*a*) Total investments given maternal energetic state. (*b*) Number of offspring given maternal energetic state. (*c*) Investment per offspring given maternal energetic state. Lines in (*a*) are products of the lines in (*b*) and (*c*). (Online version in colour.)

In our first scenario, male quality only affects the elevation of the offspring fitness function (subtracting 6 or 3 from *b*(*u*/*o*) for low- and medium-quality males, respectively), and the cost function remains the same for all males (figure 1). We find that this leads to smaller offspring when paired to high-quality males (negative DA; figures 1 and 2*c*). We also find a strong pattern of positive DA in both number of offspring and total investment (figures 1 and 2*a*,*b*), meaning that the total investment in offspring is higher when paired to a high-quality male. However, the strength of DA here is highly state-dependent and only apparent for females in moderate to high states (figure 2).

#### (ii) Male quality effects on the slope of the offspring fitness function: scenario 1B

When male quality affects the slope of the offspring fitness function (growth rate of the sigmoid function *b*(*u*/*o*), *g_m_* = {0.05, 0.125, 0.2}), we find very similar effects on DA as with changes in the elevation of the same function (scenario 1A). We therefore predict negative DA in offspring size and a relatively strong positive DA in offspring number and total investment in this case (figure 1).

#### (iii) Male quality effects on the position of the offspring fitness function: scenario 1C

When male quality affects the position of the offspring fitness function (parameter *q* in offspring fitness function changed for different males, *q_m_* = {15, 10, 5}), DA is qualitatively similar to that found in scenarios 1A and 1B, with positive DA in offspring number, negative DA in offspring size and positive DA in total investment (figure 1). Biologically, this could represent a scenario where the male invests energy in the offspring without regard to the subsequent level of female investment (i.e. a sealed bid parental investment game in which males always go first, e.g. [46]).

#### (iv) Combined effects of offspring fitness function elevation and slope: scenario 1D

In this scenario, we imagine high-quality males that will produce offspring with potentially very high fitness, but that those offspring require more energy from the female to get to that high fitness (*a_m_* = {−5, −8, −12}, *g_m_* = {0.15, 0.10, 0.05}, *k_m_* = {6, 9, 13}, *q* = 6). In this case, the slope of the offspring fitness function is then lower for high-quality males, but the upper asymptote is also higher for high-quality males (which is why we call them high quality) (figure 1). The result then becomes the opposite of what we have seen in the other scenarios (1A–C): negative DA for total investment and number of offspring, but positive DA for offspring size (figure 1).

#### (v) Constraints on the number of offspring

The trade-offs between size versus number of offspring, and between current versus future reproduction, may be affected by other factors. If we limit the maximum number of offspring, the female can produce to two for the scenario where male quality affects the elevation of the offspring fitness function (scenario 1A), we find that most of the DA effects described above disappear, and it is only at very low female states that we see the same patterns as when the number of offspring is not limited. These results are in concordance with our previous results for DA when parental care is non-depreciable [38]. Interestingly, when the number of offspring is limited and the male quality effect is via the slope of the offspring fitness function (in the same way as in scenario 1B), we find the opposite pattern to most of the other scenarios: little DA in offspring number, but negative DA in offspring size and total investment (see electronic supplementary material, S1). The difference in this result compared to scenario 1B in our previously published model [38] is obviously that the offspring fitness function we are using here has diminishing returns on parental investment, rather than being a simple linear function.

Instead of simply limiting the number of offspring, we can perhaps more realistically assume a large cost of having a higher number of offspring:3.1
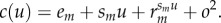


This is similar to equation (2.4) but has an additional term including *o*, the number of offspring.

In this case, we find that the patterns are qualitatively the same as when there are no limitations on the number of offspring. However, the differences in optimal reproductive decisions between females paired with different qualities of males are smaller (i.e. DA is less pronounced). There is also no interaction between the effect of male quality (either on the elevation or on the slope of the offspring fitness function) and this penalty of larger numbers of offspring (see electronic supplementary material, S1).

### (b) Male quality effects on the female cost function

The second class of male quality effects involves changes in female reproductive costs. The partner may affect how investment translates into reductions in female state either through the elevation of the cost function, the slope of the cost function or a combination of the two. We also investigate the possibility that male quality affects the position of the female on her cost function, which would be the biological equivalent of males providing nuptial gifts of different energetic value.

#### (i) Male quality effect on cost function elevation: scenario 2A

When male quality affects the elevation of the cost function (intercept *d* in cost function varies for different males, *d_m_* = {10, 5, 0}), hardly any DA is found at all, except for the effect of the additional amount of resources being made available during breeding (figure 1). The amount of DA (difference in total investment, number of offspring and investment per offspring from different quality males) is thus directly proportional to the difference in parental cost caused by the different males (see electronic supplementary material, S1). DA is also predicted to be very state-dependent and, as opposed to scenarios where males affect offspring fitness function, greater for females in lower state (see electronic supplementary material, S1).

#### (ii) Male quality effect on cost function slope: scenario 2B

When male quality affects the slope of the female cost function (parameter *s* in cost function varies for different males, *s_m_* = {0.22, 0.20, 0.18}), we find positive DA in total investment and offspring number, but no DA in investment per offspring (figure 1; electronic supplementary material, S1). Here, DA is largest for females in high energetic state.

#### (iii) Male quality effects on position of cost function: scenario 2C

When male quality affects the position of the cost function (investment *u* gives costs *c*(*u* + 5) and *c*(*u* − 5) for low- and high-quality males, respectively), our model predicts small positive DA in total investment and number of offspring (figure 1; see electronic supplementary material, S1). It also predicts mixed results for investment per offspring with alternating, but relatively small, positive and negative DA depending on maternal state and number of offspring (see electronic supplementary material, S1).

#### (iv) Male quality combined effects on cost function elevation and slope: scenario 2D

In a scenario where the high-quality male offers a low elevation (*d_m_* = {10, 5, 0}), but a steep slope (*s_m_* = {0.17, 0.2, 0.23}) for the female cost function (figure 1), we find an overall negative DA in total investment and number of offspring. However, this effect is highly state-dependent and, interestingly, for low female energetic states, the pattern is opposite: positive DA mostly manifested as no reproduction with low-quality males.

### (c) Male quality effects on both offspring fitness and female cost function: scenario 3A

Most of the results for basic male quality effects on offspring fitness function and female cost function separately are relatively straightforward and all point in the same direction (scenarios 1A–C, 2A–C). It would therefore be uninteresting to combine them in ways where high-quality males have positive effects on both components because the results plainly show that such effects would simply accumulate in the same direction. However, there are many equally realistic ways that male quality could affect female costs and offspring fitness to produce effects that are more complex. We have chosen to show one such scenario here, which corresponds to a situation where high-quality males provide genetic benefits for the offspring, but also provide less protection for their females (because of more extra-pair matings) or even costs in the form of sexually transmitted diseases. In this scenario, therefore, high-quality males have a positive effect on the slope of the offspring fitness function (*g_m_* = {0.1, 0.15, 0.2}), but a negative effect on the elevation of the female cost curve; that is, costs are higher with high-quality males (+5 or +10 added to *c*(*u*) for medium- and high-quality males, respectively). Results from this scenario are relatively intuitive and show that females should then invest more per offspring with low-quality males (i.e. negative DA in offspring size), but have more offspring with high-quality males (i.e. positive DA in offspring number; figure 1). If the positive and negative effects are balanced, such as in this example, total investment will be very similar for all male qualities (figure 1) and in essence only depend on maternal energetic state (electronic supplementary material, S1). There was a slight tendency for negative DA in total investment for low maternal states, and positive DA in total investment for high maternal states (electronic supplementary material, S1).

## 4. Discussion

Our model shows that the main patterns of DA are not theoretically difficult to predict for a specific population if one is able to (i) disentangle whether partner quality affects the offspring fitness benefits or the parental investment costs, and (ii) understand how partner quality affects the shape of the offspring fitness function. In general, we predict that higher total investment and number of offspring when paired with high-quality partners should be a relatively common pattern under a range of scenarios. Females should also always base their investment per offspring upon maximizing the marginal value per unit of investment [39,41]. Therefore, when the male quality effect is on the offspring fitness function (scenarios 1A–C), we commonly expect negative DA (lower investment in offspring from high-quality males) in investment per offspring (offspring size). To understand this negative DA in offspring size, it can be helpful to look at where the tangent from origin hits the offspring fitness function (figure 1, scenarios 1A–C). This happens at lower levels of investment for offspring of high-quality males, but also allows females to receive more fitness per invested unit of energy when mated to high-quality males. This explains the positive DA for total reproductive investment in these baseline scenarios, but this investment is best divided up between more offspring each time. It is only in scenario 1D, when the partner affects both the slope and the maximum value of the offspring fitness, that we find positive DA (higher investment in offspring from high-quality males) in offspring size. When partner quality affects female costs (scenarios 2A–C), all of the positive DA we find in total reproductive investment is coming from an increased number of offspring with high-quality males, because male quality does not affect the optimal offspring size.

Our results generally agree well with previous models where there is overlap in the biological scenario. Kindsvater & Alonzo [39] chose to focus on parameter values in the offspring fitness function and they relate all predicted changes in optimal investment to those parameters rather than to partner quality. One exception is their nuptial gift scenario, which corresponds to our scenario 2C where male quality affects the position of the female cost curve. The scenario where we investigate male quality effects on the slope of the offspring fitness function (1B) is in many ways similar to the rest of the scenarios presented in Kindsvater & Alonzo [39], and none of our results in this regard directly contradict the predictions made by their model. However, we also show that there should be additional trade-offs, because in our model we allow a change of partners between breeding seasons. Therefore, we not only find DA in offspring number and size, but also in total reproductive investment as well.

Our predictions for partner effects on offspring fitness (scenarios 1A–C) differ from those derived for cases when females invest in only one offspring at a time or investment is non-depreciable [38]. Our current model predicts that similar results can be found even when male quality has no effect on the slope of the offspring fitness function, but only on its elevation or its position. Because females can divide their resources between as many offspring as they like in the current model, the decision concerning the size of offspring is decoupled from the trade-off between current and future reproduction. When these trade-offs are decoupled, females are able to maximize fitness per investment independently of total investment (given large enough energetic reserves relative to optimal offspring size). If females are limited to only one offspring or care is non-depreciable, female fitness per investment is decided based only upon total investment in the current reproductive attempt [38], and therefore cannot be decoupled from the trade-off with future reproduction. We investigated two intermediate scenarios to explore this difference in detail (see above, ‘Constraints on the number of offspring’). When we limit the female to only two offspring, we unsurprisingly find DA results very similar to the non-depreciable care model [38]. By contrast, introducing a cost that increases with the number of offspring does not have such decisive effect, and the results are instead similar to the effect in the baseline scenario and clearly different from the non-depreciable care model [38].

Our results underline the importance of understanding the exact effects of partner quality on the costs and benefits in order to produce robust predictions for DA in all components of parental investment. Few empirical studies to date have investigated this and it is therefore hard to validate our model with existing data. A review of DA in birds suggests that biparental species tend to show positive DA in offspring number, whereas species with female-only care tend to show positive DA in egg size [47]. This is in general agreement with our results, if we make the sensible assumption that male quality in biparental systems will tend to also include effects on female costs, for example via territory qualities or a reduced need to fend off predators. In the systems with female-only care, we expect to find more polygynous species where male quality effects on offspring fitness are likely to be similar to our combined effects scenario (1D).

Scenario 1D (figure 1) illustrates that if offspring from high-quality males have the potential to obtain a higher fitness eventually, but require a larger investment to reach that potential fitness, females should invest more in each offspring from high-quality males and have fewer offspring. This is a realistic scenario, for example in polygynous mating systems with high reproductive skew, where individuals with the highest potential fitness may have sexually selected ornaments that are genetically determined and costly to grow. The pattern is akin to the differential investment in sons over daughters seen in many polygynous species [48,49]. Studies on gallinaceous birds, such as peafowl [18,36], quail [50] and mallards [51,52], as well as other species with high reproductive skew (the lekking lance-tailed manakin [37]), tend to show positive DA in per-offspring investment but no DA in clutch size. Only the first part of these results aligns with our predictions from scenario 1D. In this scenario, we also expect negative DA in clutch size, which is not seen in these studies. There are several plausible explanations for this discrepancy, such as a prohibitively large cost of producing more offspring when mated with poor-quality males, an alternative shape to the maternal cost function or possibly another trade-off not accounted for in our model. Detailed empirical examination of the effect of male quality on both offspring fitness and maternal cost functions alongside the modelling framework presented here should reveal an explanation for the patterns of DA observed in maternal investments in these birds.

It is not only reproductive skew systems where model scenario 1D may be useful. Galeotti *et al*. [40] showed that female crayfish laid larger but fewer eggs when paired with small males with relatively large chelae, and more but smaller eggs for large males with relatively small chelae, but there were no significant effects of male quality on total clutch weight. It is plausible here that offspring of younger (smaller) higher quality males can provide females with higher fitness returns (due to higher mating success) later in life, but that they demand more resources from her during the extended period of uniparental care [53]. Interestingly, Caro *et al.* [54] show that parent birds tend to feed offspring of higher quality more than their lower-quality siblings when the environment is poor and there is a chance of adaptive brood reduction, while the reverse is true for good environments where it is possible to raise all the offspring successfully and so offspring in most need are preferentially fed. One could also imagine systems with alternative life-history strategies, in which some ‘low-quality’ individuals have low average fitness, but also require less parental investment to produce, and so the marginal fitness gains for parents could be similar for both types, as described in the patterns predicted by Lessells [55].

Another commonly reported positive DA pattern in empirical studies is an increase in offspring number, with no effect (or no mention of an effect) on per-offspring investment (e.g. [21–24]). When interpreting these findings, we must consider that in natural systems, the possibilities for DA in the different components of reproduction may be constrained if the number of offspring or per-offspring investment is decided at some time before the female meets her partner. In such cases, our current model would predict this observed pattern of positive DA only if male quality affects the reproductive costs of the mother (scenarios 2A–C). Our results for these types of scenarios are also well aligned with previous theoretical models of DA in non-depreciable care situations [38].

DA with respect to mate quality may be seen as just a special case of a more general theory of reproductive allocation in different environments [6]. How a parent should adjust their allocation of investment in offspring size versus number and current versus future offspring is a topic that has been extensively studied [1,56,57]. For example, egg sizes differ between environments in nature [58] and it has been shown that the relationship between egg size and correlates of offspring fitness can differ according to population density [59,60], various abiotic environmental variables [61], host species differences [62] and probably due to interactions with other species. Viewing our results in a more general light suggests that the male quality effects we show on maternal investment, in terms of changes in the offspring fitness or maternal cost functions, could also predict optimal maternal responses to variation in population density or any other environmental factor important to reproduction. In general, empirical studies show a larger environmental effect on variation in fecundity than on offspring size [63,64]. This matches the predictions of our model if the main environmental effects operate through the costs associated with reproduction rather than via changes in offspring fitness benefits, which seems reasonable.

In conclusion, we challenge empiricists to test our model by assessing partner effects on both total reproductive effort and on offspring size versus number in the same systems. Crucial information is also required concerning likely partner effects on offspring fitness functions versus parental cost functions to allow comparisons against the appropriate DA models predictions (figure 1). Lastly, interpretations of empirical results must take into account that the direction of any DA is expected to differ for the different measures of parental investment (e.g. per offspring versus per brood).

## Supplementary Material

Full results

## Data accessibility

The R code is available from the Dryad Digital Repository: http://dx.doi.org/10.5061/dryad.3kf1913 [65].
