# Supplementary material for: Differential allocation of parental investment and the trade-off between size and number of offspring
Source: Proc Biol Sci. 2018 Aug 1;285(1884):20181074. doi: 10.1098/rspb.2018.1074 (PMC6111174; doi:10.1098/rspb.2018.1074)
Supplement: Full results [file rspb20181074supp1.pdf]

Complete results from the stochastic dynamic model and subsequent simulations

Supplementary material for:

Differential allocation of parental investment and the trade-off between size and number of offspring

Proceedings of the Royal Society B. 2018. doi: 10.1098/rspb.2018.1074

Irja Ida Ratikainen, Thomas Ray Haaland and Jonathan Wright

In this document, we first present a table showing all model parameters, their explanations and baseline values, then we present graphically all results from all scenarios described in Ratikainen et. al. (doi: 10.1098/rspb.2018.1074). Descriptions of the scenarios and comments on the results can be found in the main manuscript.

Table of Contents

Table of model parameters 2
1: Male quality effects on offspring fitness function 3
Scenario 1A: Male quality effects on the elevation of the offspring fitness function 3
Scenario 1B: Male quality effects on the slope of the offspring fitness function........ 7
Scenario 1C: Male quality effects on the position of the offspring fitness function 13
Scenario 1D: Combined effects of offspring fitness function elevation and slope. 15
2: Male quality effects on the female cost function 17
Scenario 2A: Male quality effect on cost function elevation 17
Scenario 2B: Male quality effect on cost function slope 19
Scenario 2C: Male quality effects on position of cost function 21
Scenario 2D: Male quality combined effects on cost function elevation and slope 23
Scenario 3A: Male quality effects on both offspring fitness and female cost function 25

Table of model parameters

Table 1: Description of functions, variables and parameters in the stochastic dynamic model. The values shown are the baseline values used in all model scenarios except where otherwise stated.

b	Benefits of reproductive investment for the female; the offspring fitness function, eq. 3.
c	Costs of reproductive investment for the female; the female cost function, eq. 4, or 4b.
u	Total reproductive investment by the female (or male).
o	Number of offspring. o_{max} = 15 in baseline model runs.
x	Female energetic state, with x_{max} = 100 and x_{min} = 3 and increments of 1.<br>Female dies if $x < x_{min}$.
α	Survival probability. Baseline: 0.8.
λ	Probability of finding food. Baseline: 0.8.
y	Energetic value of food. Baseline: 30.
T	Maximum life span, set to 100.
m	Male qualities. m = 1 is a poor male, m = 2 is medium, m = 3 is good.
N_m	Number of male qualities, set to 3.
$\boldsymbol{P}$	Probability distribution of meeting the different male types. P_m is a component of vector $\boldsymbol{P}$.<br>We examined $\boldsymbol{P}$ = {0.3,0.4,0.3} (baseline), $\boldsymbol{P}$ = {0.6,0.2,0.2} and $\boldsymbol{P}$ = {0.1,0.6,0.3}.
a_m	Offspring fitness function lower asymptote. Baseline -11.
k_m	Offspring fitness function upper asymptote. Baseline 11
g_m	Offspring fitness function growth rate. Baseline 0.2.
q_m	Offspring fitness function inflection point. Baseline 5.
d_m	Cost function intercept. Baseline 0.
r_m	Cost function shape parameter. Baseline 1.3.
s_m	Cost function slope parameter. Baseline 0.2.

1: Male quality effects on offspring fitness function

Scenario 1A: Male quality effects on the elevation of the offspring fitness function

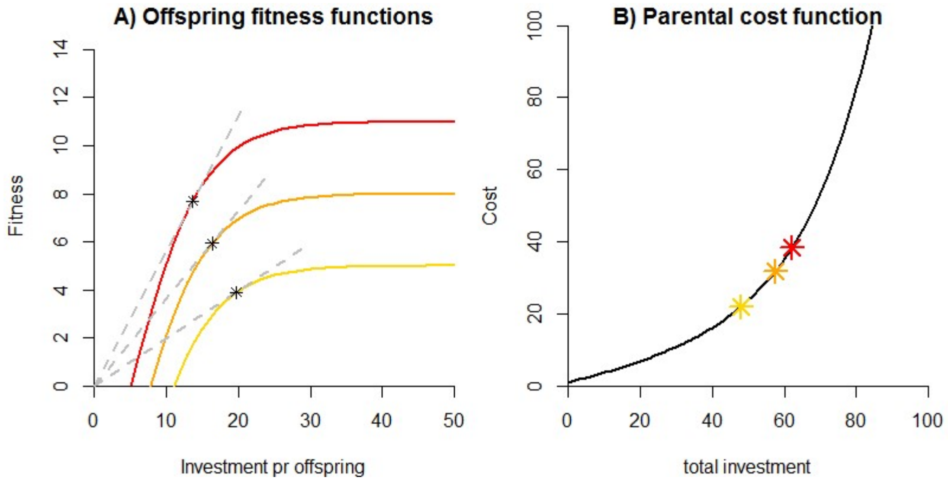

Figure S1. Scenario 1A. A) Offspring fitness functions for different quality males dependent on maternal investment per offspring. Tangents from origin to each of the fitness functions are drawn in grey dotted lines. The stars indicate the average of the optimal investment for the 80 highest maternal states for the three different male qualities. B) Maternal cost function given total reproductive investment. Stars indicate the cost for the optimal investment of females paired with the three different quality males (yellow: low quality, orange: medium quality, red: high quality).

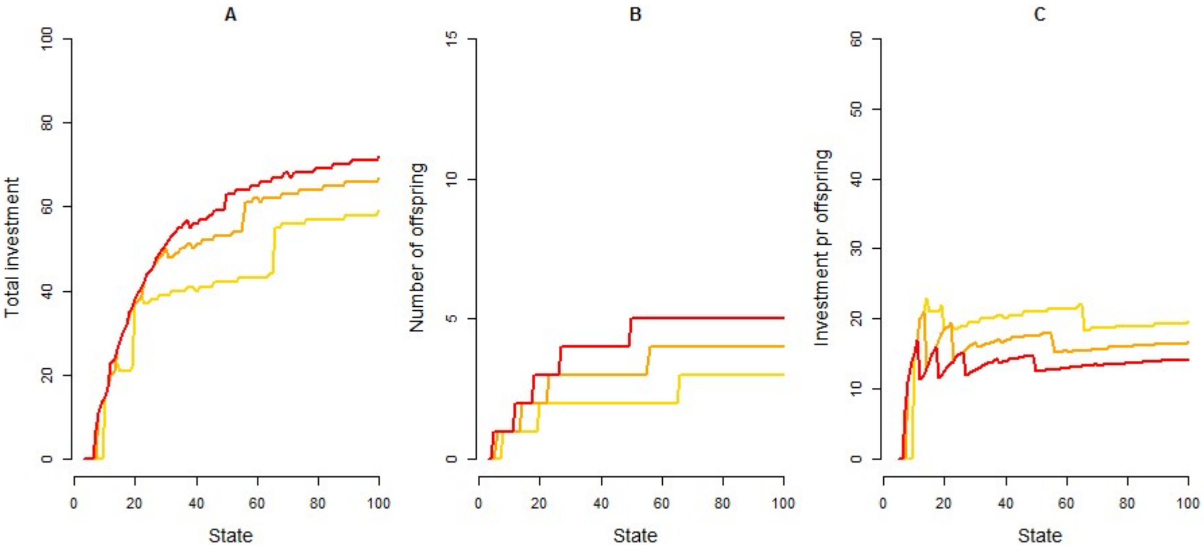

Figure S2 (Presented as figure 2 in main manuscript). Plots showing state dependent results from the stochastic dynamic optimization model for scenario 1A. Each panel shows the different optimal investments along the axis of female energetic state (x-axis). Yellow lines correspond to low quality males, orange medium quality and red high quality males. A) Total investments given maternal energetic state. B) Number of offspring given maternal energetic state. C) Investment per offspring given maternal energetic state. Lines in A) are products of the lines in B) and C).

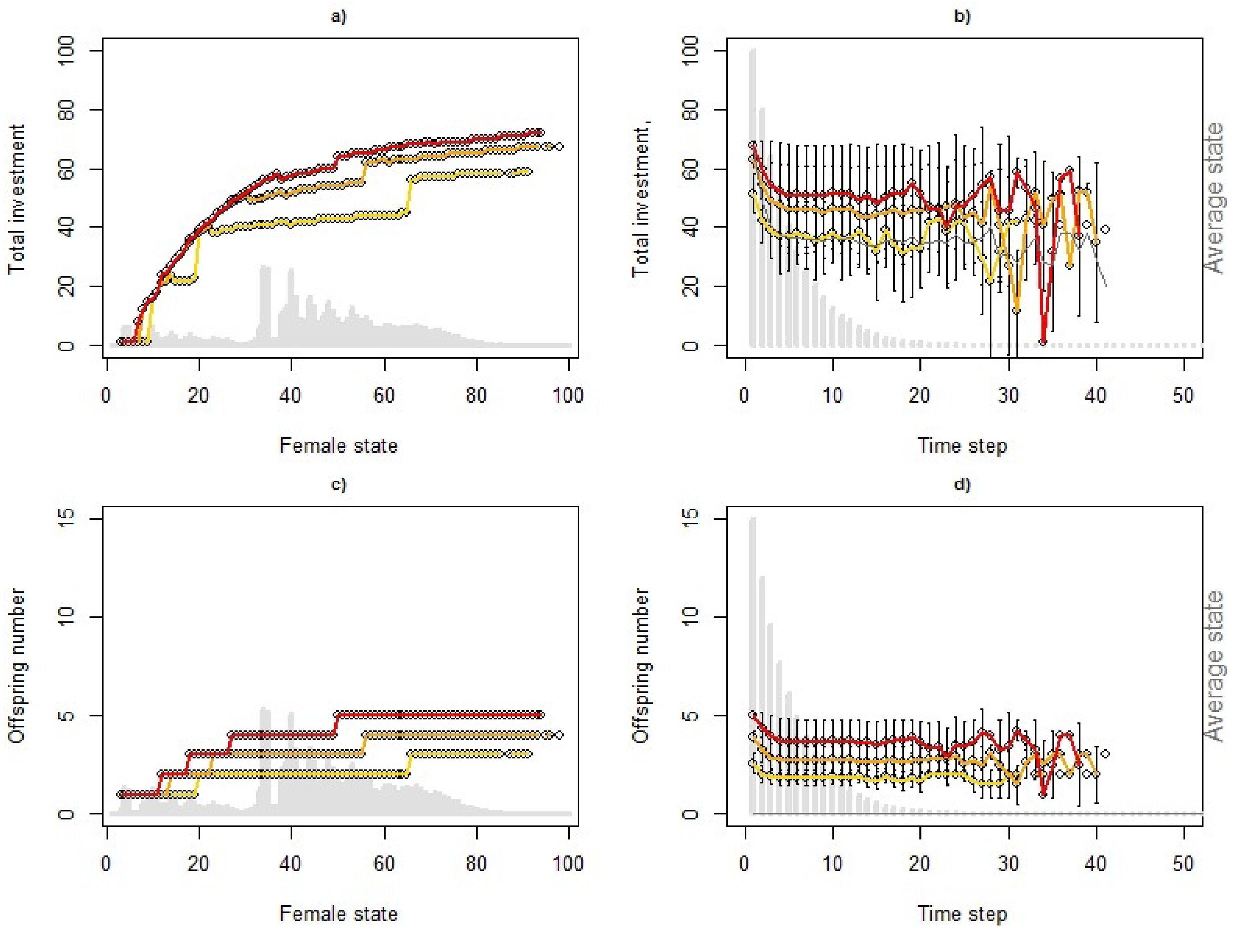

Figure S3. Results from simulation model for scenario 1A. In all panels red lines represent investments in offspring from high quality males, orange for medium quality males and yellow for low quality males. Grey bars in the background show distributions of females in different states (a and c) and over different time steps (b and d). Black error bars show standard deviations. a) Total investment in relation to maternal energetic state. b) Total investment over time. c) Number of offspring in relation to maternal energetic state. d) Number of offspring over time.

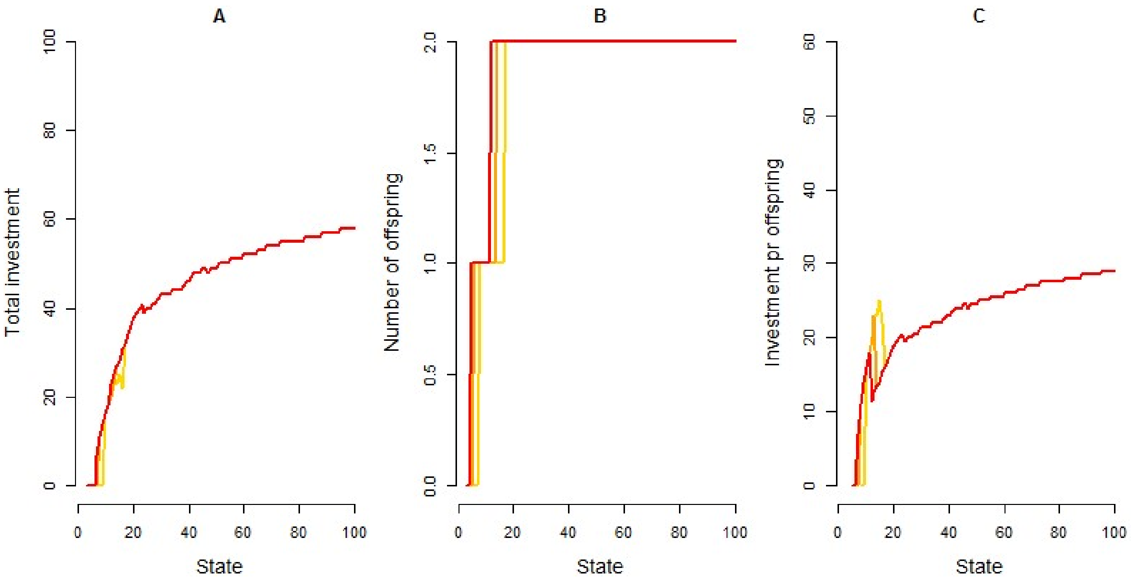

Figure S4. Plots showing state dependent results from stochastic dynamic optimization model for scenario 1A, but limited to maximum two offspring. Each panel shows the different optimal investments along the axis of female energetic state (x-axis). Yellow lines correspond to low quality males, orange medium quality and red high quality males. A) Total investments given maternal energetic state. B) Number of offspring given maternal energetic state. C) Investment per offspring given maternal energetic state. Lines in A) are products of the lines in B) and C).

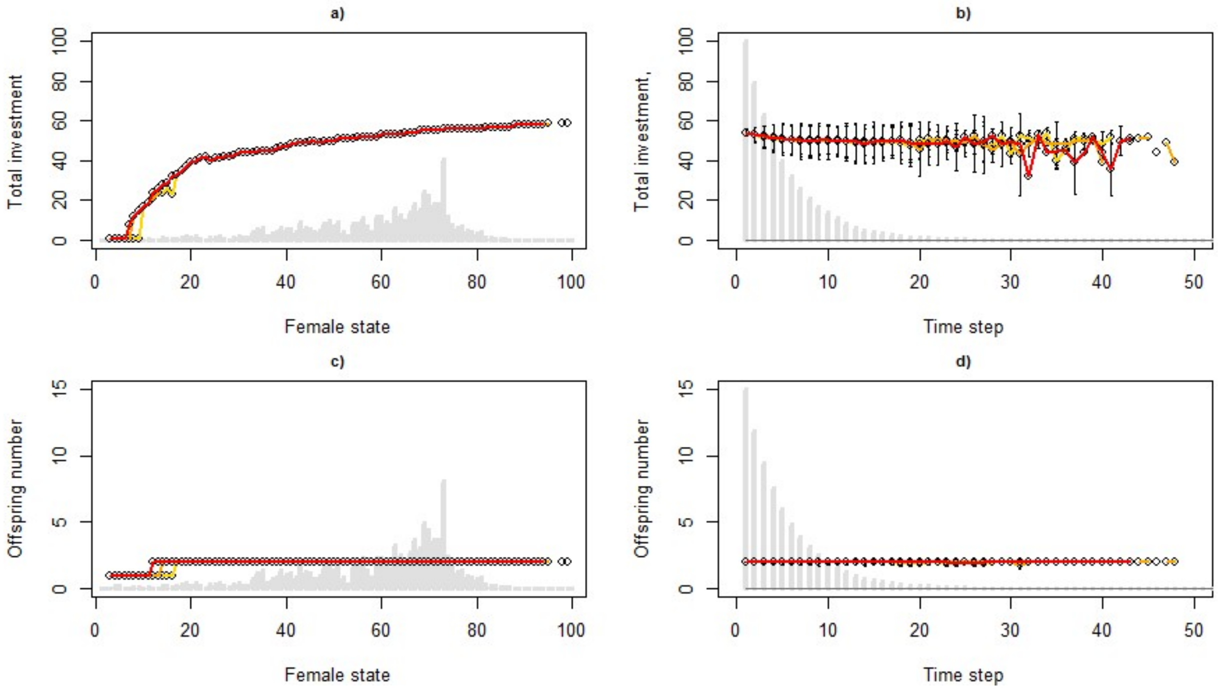

Figure S5. Results from simulation model for scenario 1A with maximum two offspring. In all panels red lines represent investments in offspring from high quality males, orange for medium quality males and yellow for low quality males. Grey bars in the background show distributions of females in different states (a and c) and over different time steps (b and d). Black error bars show standard deviations. a) Total investment in relation to maternal energetic state. b) Total investment over time. c) Number of offspring in relation to maternal energetic state. d) Number of offspring over time.

Scenario 1B: Male quality effects on the slope of the offspring fitness function

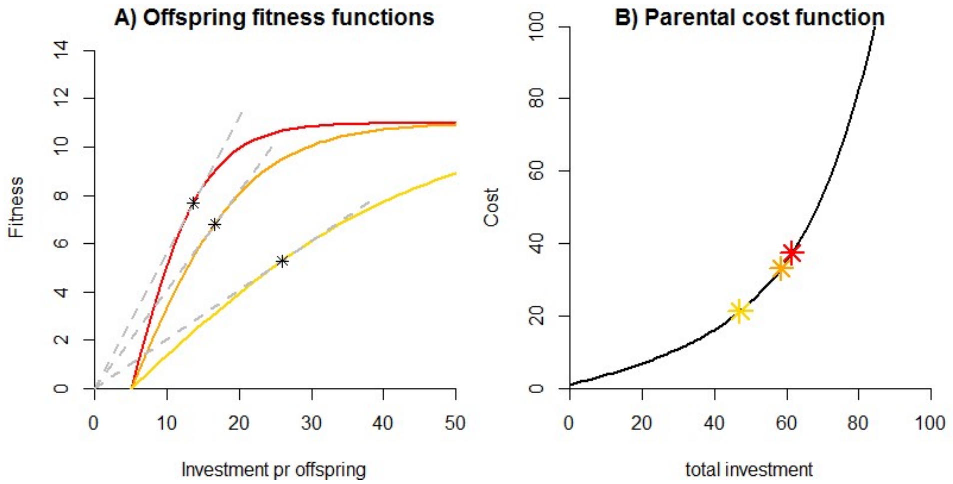

Figure S6. Scenario 1B. A) Offspring fitness functions for different quality males dependent on maternal investment per offspring. Tangents from origin to each of the fitness functions are drawn in grey dotted lines. The stars indicate the average of the optimal investment for the 80 highest maternal states for the three different male qualities from the stochastic dynamic optimization model. B) Maternal cost function given total reproductive investment. Stars indicate the cost for the optimal investment of females paired with the three different quality males (yellow: low quality, orange: medium quality, red: high quality) from the stochastic dynamic model.

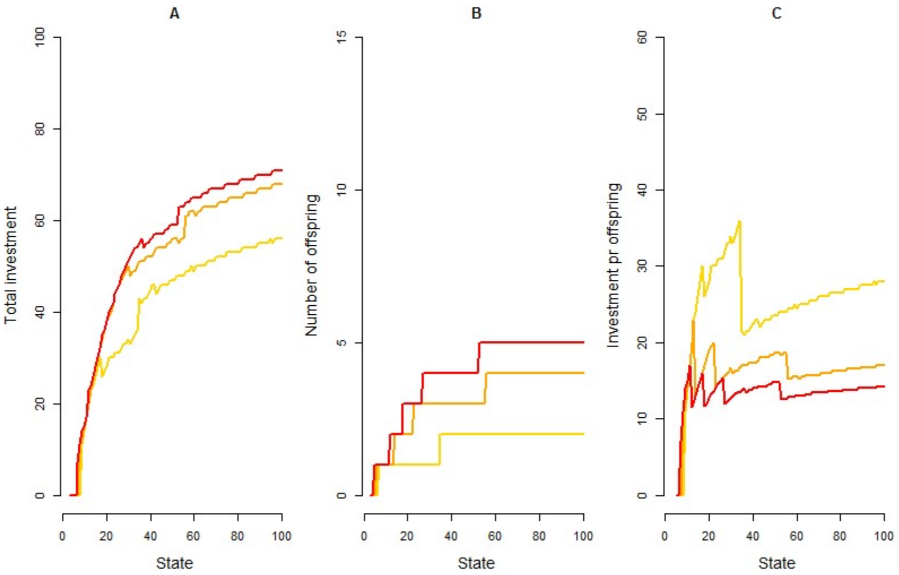

Figure S7. Plots showing state dependent results from the stochastic dynamic optimization model for scenario 1B. Each panel shows the different optimal investments along the axis of female energetic state (x-axis). Yellow lines correspond to low quality males, orange medium quality and red high quality males. A) Total investments given maternal energetic state. B) Number of offspring given maternal energetic state. C) Investment per offspring given maternal energetic state. Lines in A) are products of the lines in B) and C).

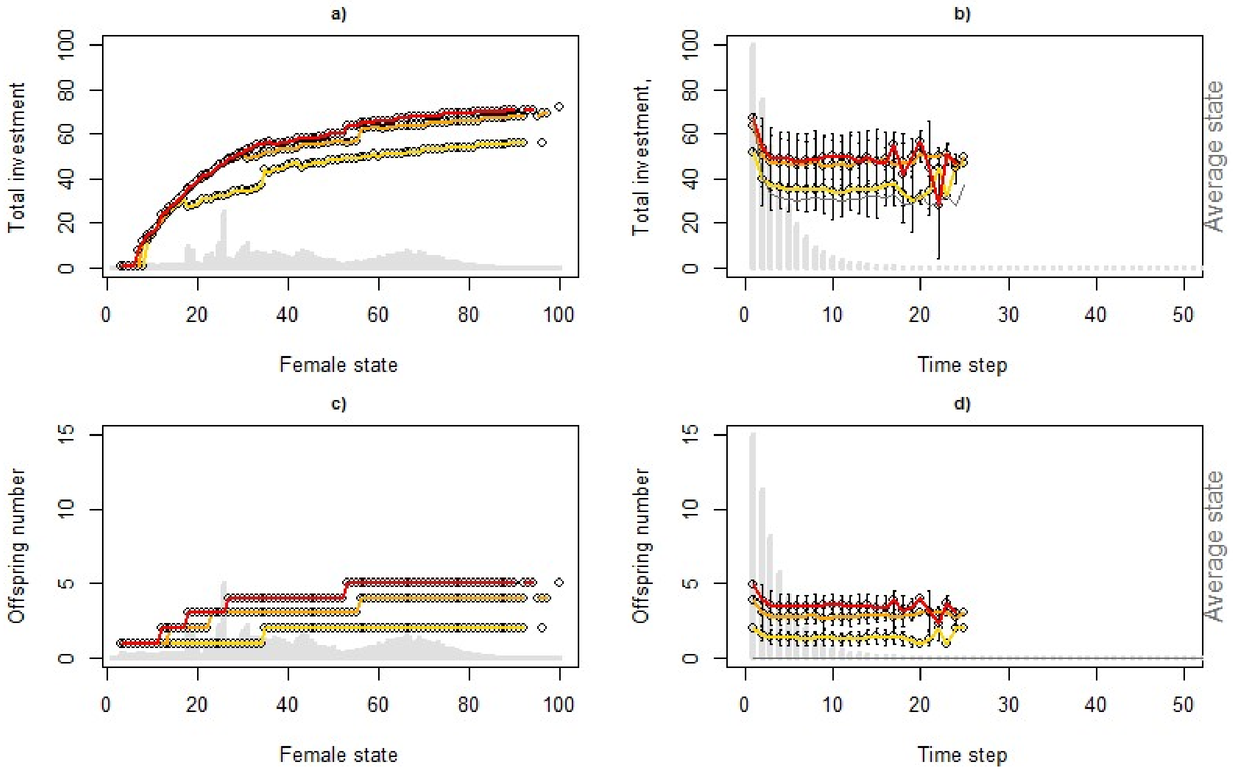

Figure S8. Results from simulation model for scenario 1B. In all panels red lines represent investments in offspring from high quality males, orange for medium quality males and yellow for low quality males. Grey bars in the background show distributions of females in different states (a and c) and over different time steps (b and d). Black error bars show standard deviations. a) Total investment in relation to maternal energetic state. b) Total investment over time. c) Number of offspring in relation to maternal energetic state. d) Number of offspring over time.

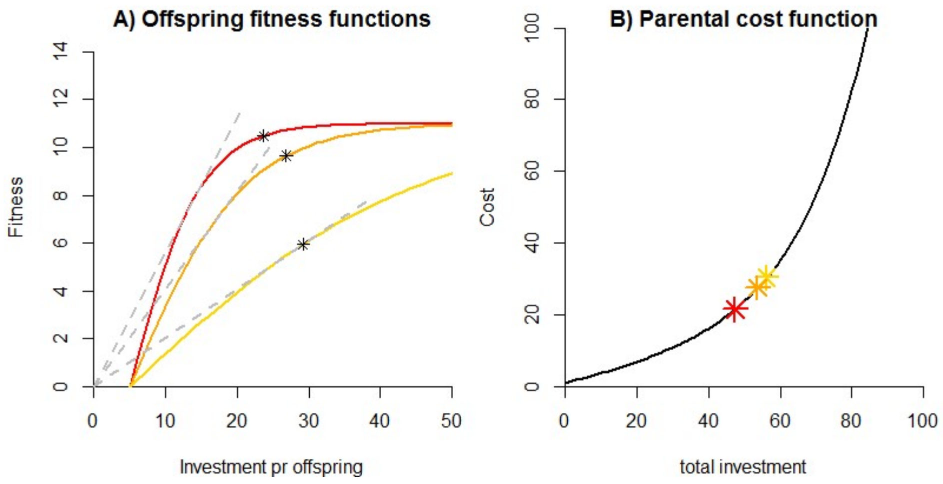

Figure S9. Scenario 1B with maximum two offspring. A) Offspring fitness functions for different quality males dependent on maternal investment per offspring. Tangents from origin to each of the fitness functions are drawn in grey dotted lines. The stars indicate the average of the optimal investment for the 80 highest maternal states for the three different male qualities. Note that this scenario with a maximum of two offspring is the only case where the average optimal investments are not where the tangent first meets the curve of the fitness functions. B) Maternal cost function given total reproductive investment. Stars indicate the cost for the optimal investment of females paired with the three different quality males (yellow: low quality, orange: medium quality, red: high quality).

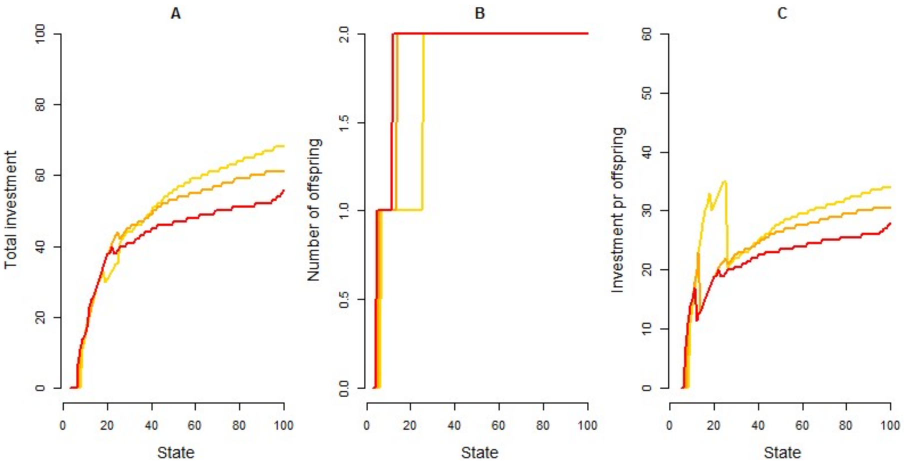

Figure S10. Plots showing state dependent results from stochastic dynamic optimization model for scenario 1B with maximum two offspring. Each panel shows the different optimal investments along the axis of female energetic state (x-axis). Yellow lines correspond to low quality males, orange medium quality and red high quality males. A) Total investments given maternal energetic state. B) Number of offspring given maternal energetic state. C) Investment per offspring given maternal energetic state. Lines in A) are products of the lines in B) and C).

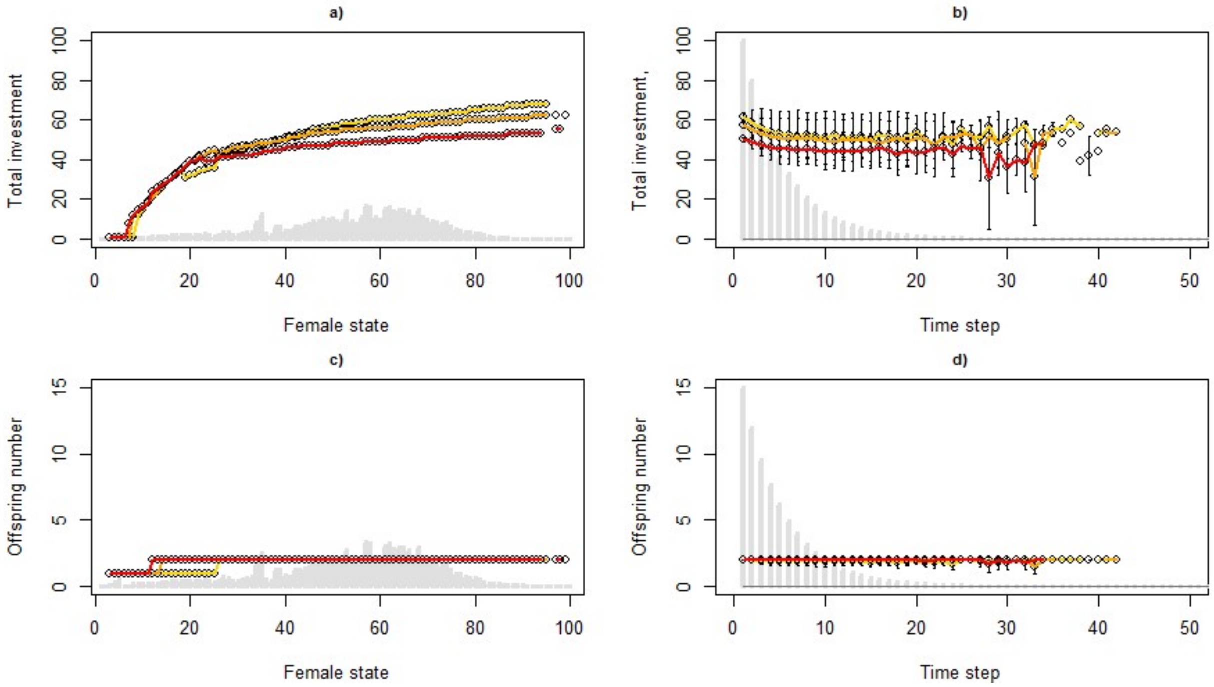

Figure S11. Results from simulation model for scenario 1B with a limitation of maximum two offspring in each reproductive event. In all panels red lines represent investments in offspring from high quality males, orange for medium quality males and yellow for low quality males. Grey bars in the background show distributions of females in different states (a and c) and over different time steps (b and d). Black error bars show standard deviations. a) Total investment in relation to maternal energetic state. b) Total investment over time. c) Number of offspring in relation to maternal energetic state. d) Number of offspring over time.

Penalty on the number of offspring

In this scenario the costs increase with the of number of offspring.

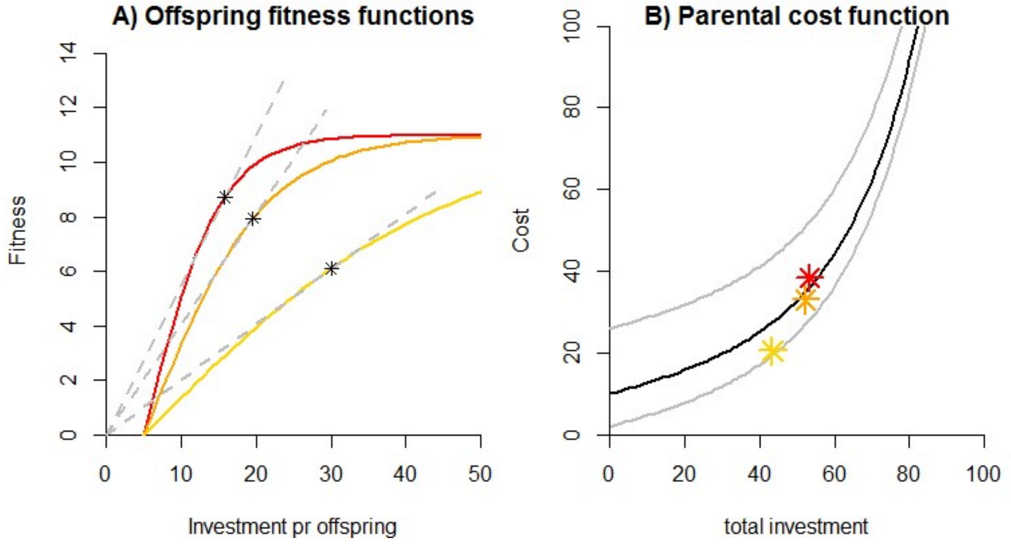

Figure S12. Scenario 1B with additional costs with increasing number of offspring. A) Offspring fitness functions for different quality males dependent on maternal investment per offspring. The stars indicate the average of the optimal investment for the 80 highest maternal states for the three different male qualities from the stochastic dynamic optimization model. Lines from origin to each of the optimal investments are drawn in grey dotted lines. Note that the slopes of these are slightly lower than the slopes of the tangents from the origin to the fitness functions. B) Maternal cost functions given total reproductive investment for 1 (lower grey line), 3 (black line) and 5 (upper grey line) offspring. Stars indicate the cost for the optimal investment of females paired with the three different quality males (yellow: low quality, orange: medium quality, red: high quality) from the stochastic dynamic model based on an average for the 80 highest maternal states.

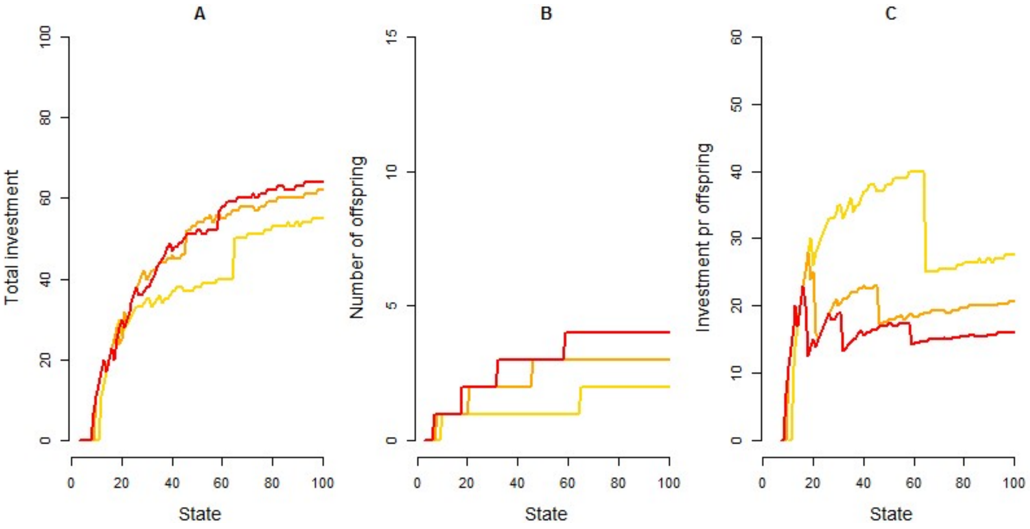

Figure S13. Plots showing state dependent results from the stochastic dynamic optimization model for scenario 1B with additional costs with increasing number of offspring. Each panel shows the different optimal investments along the axis of female energetic state (x-axis). Yellow lines correspond to low

quality males, orange medium quality and red high quality males. A) Total investments given maternal energetic state. B) Number of offspring given maternal energetic state. C) Investment per offspring given maternal energetic state. Lines in A) are products of the lines in B) and C).

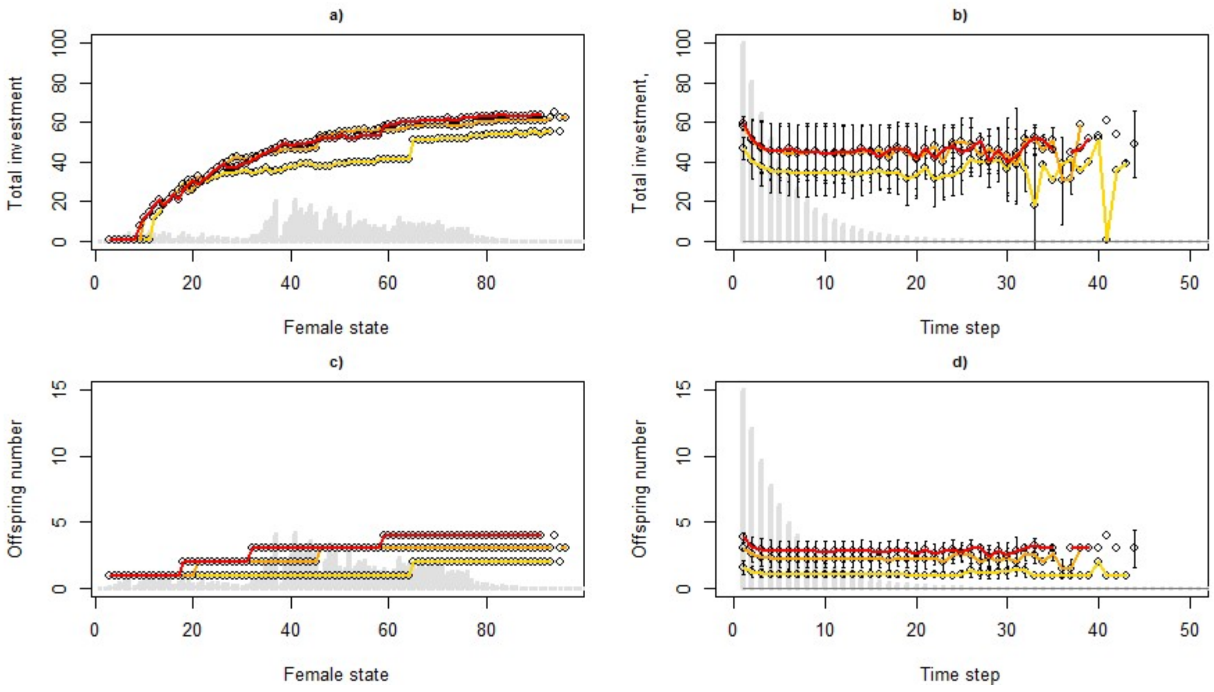

Figure S14. Results from simulation model for scenario 1B with additional costs with increasing number of offspring. In all panels red lines represent investments in offspring from high quality males, orange for medium quality males and yellow for low quality males. Grey bars in the background show distributions of females in different states (a and c) and over different time steps (b and d). Black error bars show standard deviations. a) Total investment in relation to maternal energetic state. b) Total investment over time. c) Number of offspring in relation to maternal energetic state. d) Number of offspring over time.

Scenario 1C: Male quality effects on the position of the offspring fitness function

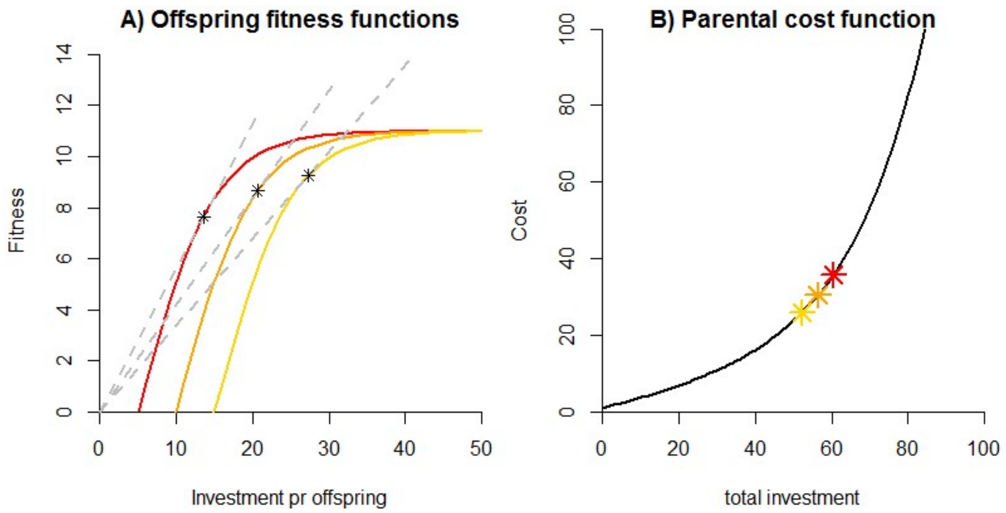

Figure S15. Scenario 1C. A) Offspring fitness functions for different quality males dependent on maternal investment per offspring. Tangents from origin to each of the fitness functions are drawn in grey dotted lines. The stars indicate the average of the optimal investment for the 80 highest maternal states for the three different male qualities from the stochastic dynamic optimization model. B) Maternal cost function given total reproductive investment. Stars indicate the cost for the optimal investment of females paired with the three different quality males (yellow: low quality, orange: medium quality, red: high quality) from the stochastic dynamic model.

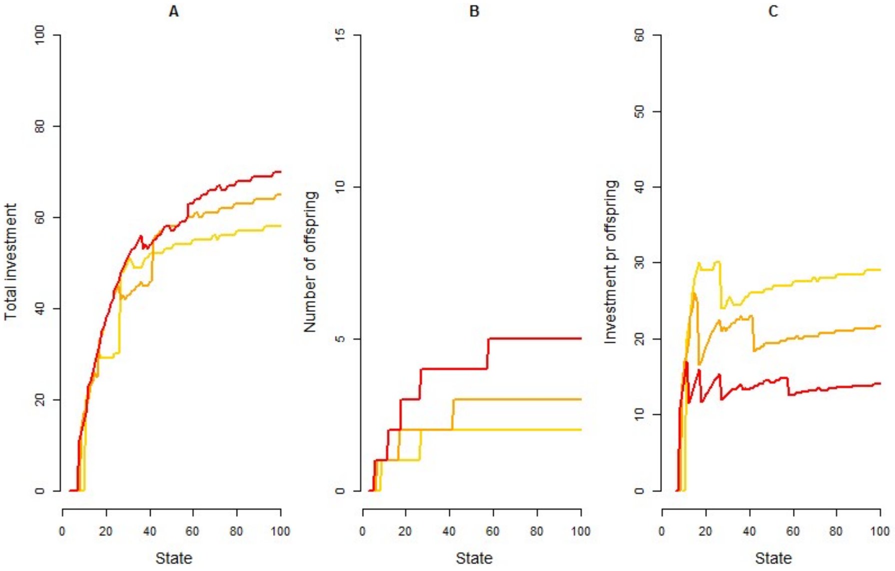

Figure S16. Plots showing state dependent results from the stochastic dynamic optimization model for scenario 1C. Each panel shows the different optimal investments along the axis of female energetic state (x-axis). Yellow lines correspond to low quality males, orange medium quality and red high quality males. A) Total investments given maternal energetic state. B) Number of offspring given maternal energetic state. C) Investment per offspring given maternal energetic state. Lines in A) are products of the lines in B) and C).

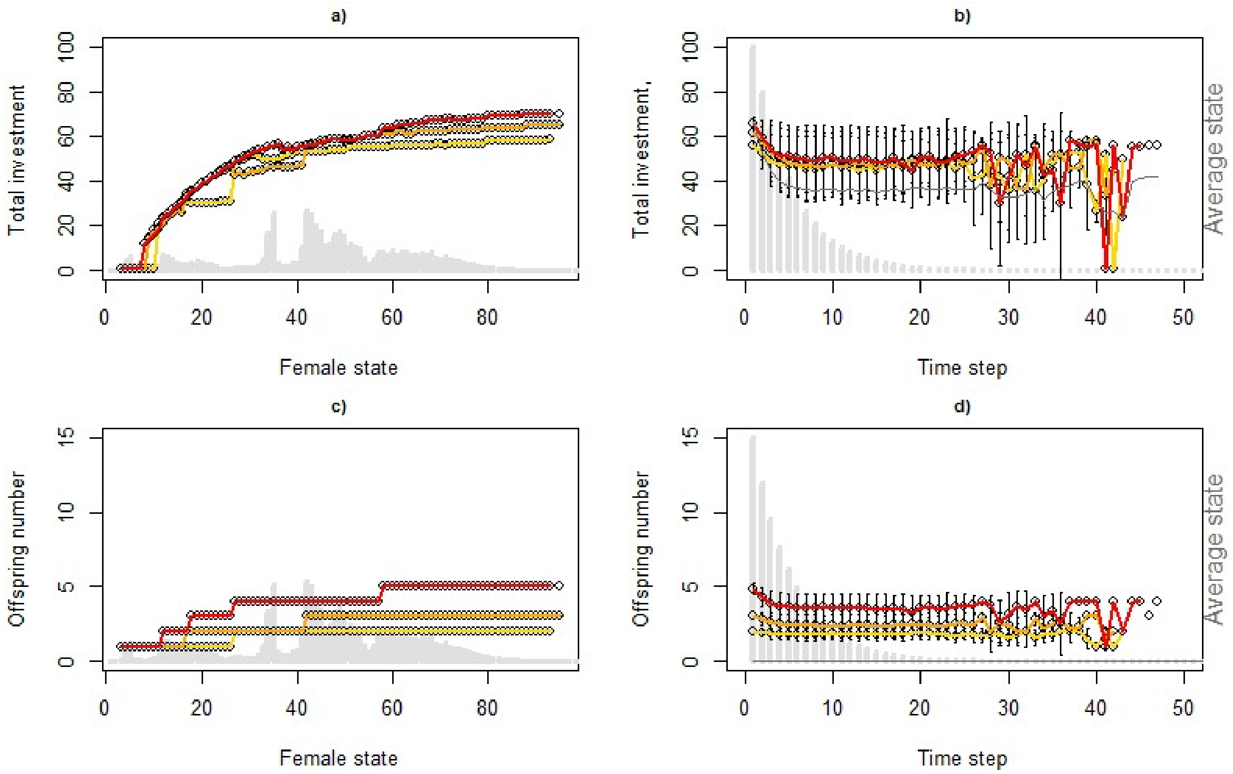

Figure S17. Results from simulation model for scenario 1C. In all panels red lines represent investments in offspring from high quality males, orange for medium quality males and yellow for low quality males. Grey bars in the background show distributions of females in different states (a and c) and over different time steps (b and d). Black error bars show standard deviations. a) Total investment in relation to maternal energetic state. b) Total investment over time. c) Number of offspring in relation to maternal energetic state. d) Number of offspring over time.

Scenario 1D: Combined effects of offspring fitness function elevation and slope.

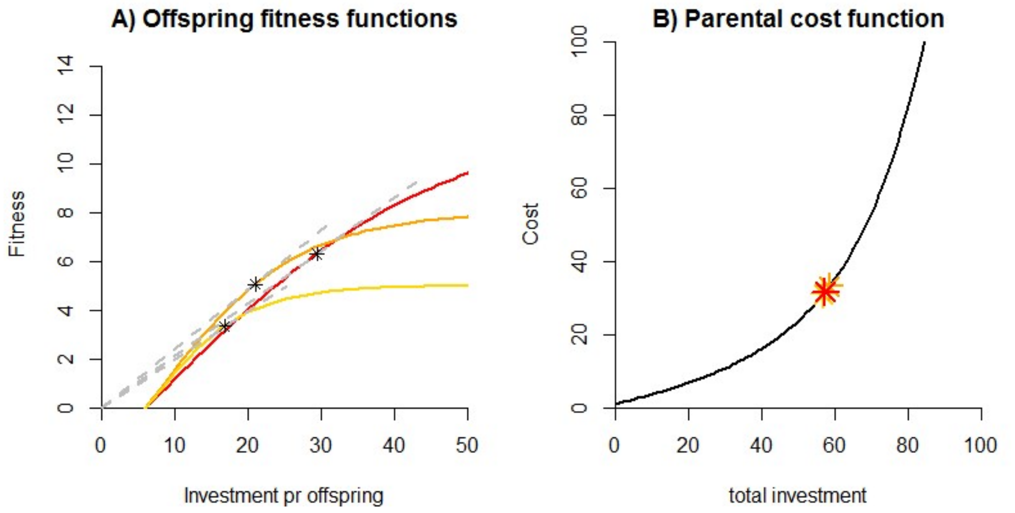

Figure S18. Scenario 1D. A) Offspring fitness functions for different quality males dependent on maternal investment per offspring. Tangents from origin to each of the fitness functions are drawn in grey dotted lines. The stars indicate the average of the optimal investment for the 80 highest maternal states for the three different male qualities from the stochastic dynamic optimization model. B) Maternal cost function given total reproductive investment. Stars indicate the cost for the optimal investment of females paired with the three different quality males (yellow: low quality, orange: medium quality, red: high quality) from the stochastic dynamic model.

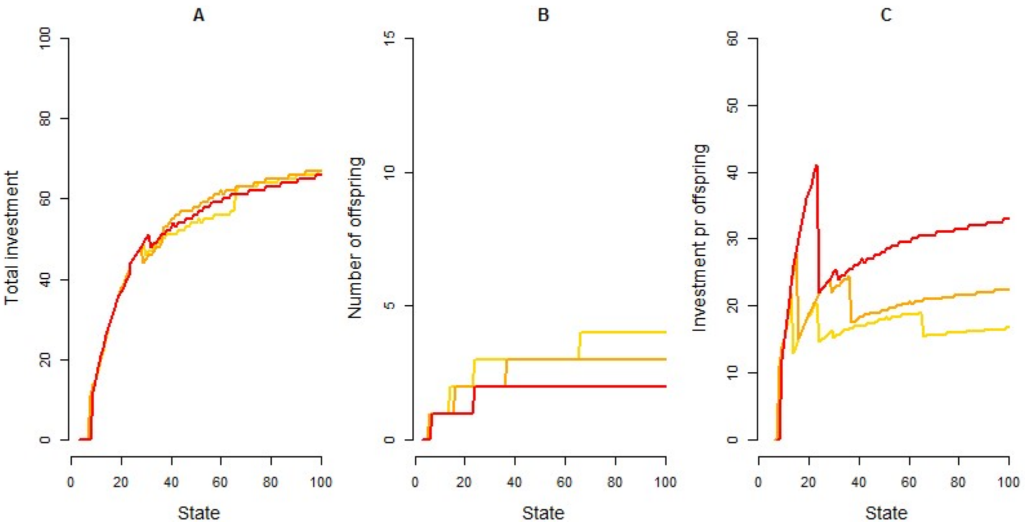

Figure S19. Plots showing state dependent results from the stochastic dynamic optimization model for scenario 1D. Each panel shows the different optimal investments along the axis of female energetic state (x-axis). Yellow lines correspond to low quality males, orange medium quality and red high quality males. A) Total investments given maternal energetic state. B) Number of offspring given maternal energetic state. C) Investment per offspring given maternal energetic state. Lines in A) are products of the lines in B) and C).

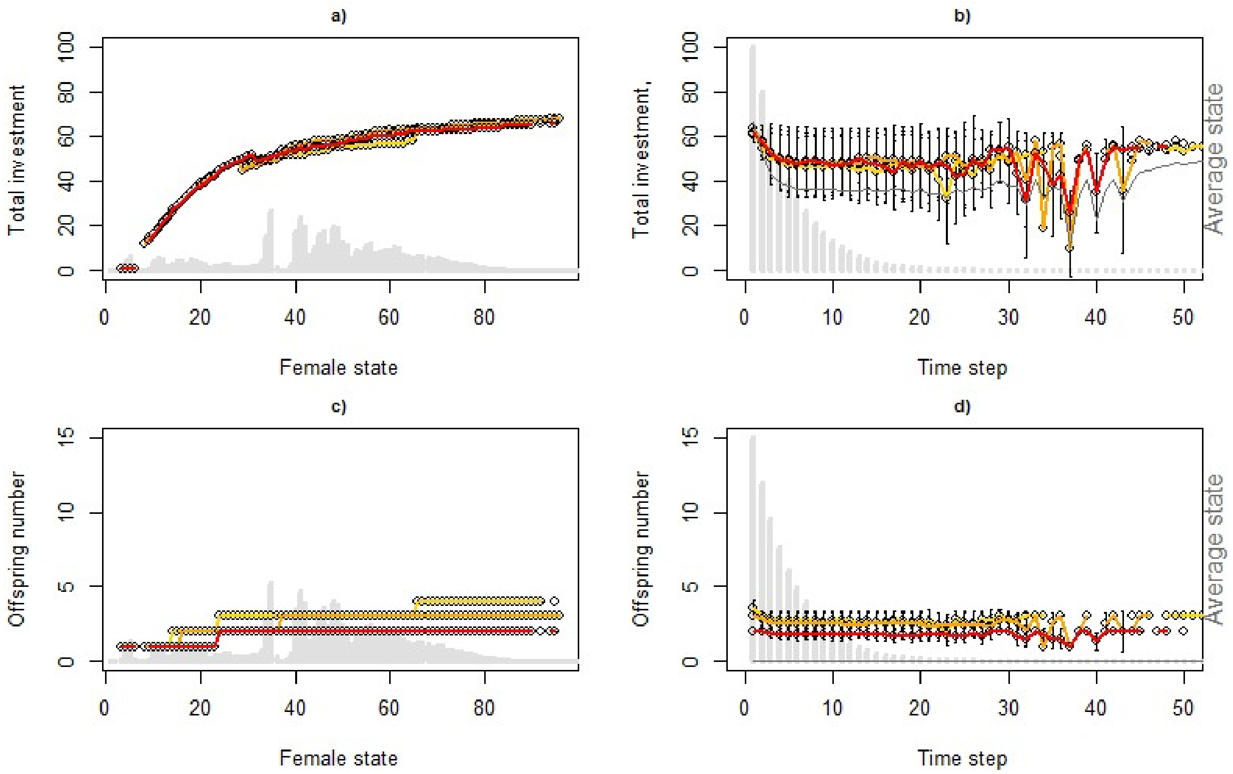

Figure S20. Results from simulation model for scenario 1D. In all panels red lines represent investments in offspring from high quality males, orange for medium quality males and yellow for low quality males. Grey bars in the background show distributions of females in different states (a and c) and over different time steps (b and d). Black error bars show standard deviations. a) Total investment in relation to maternal energetic state. b) Total investment over time. c) Number of offspring in relation to maternal energetic state. d) Number of offspring over time.

2: Male quality effects on the female cost function

Scenario 2A: Male quality effect on cost function elevation

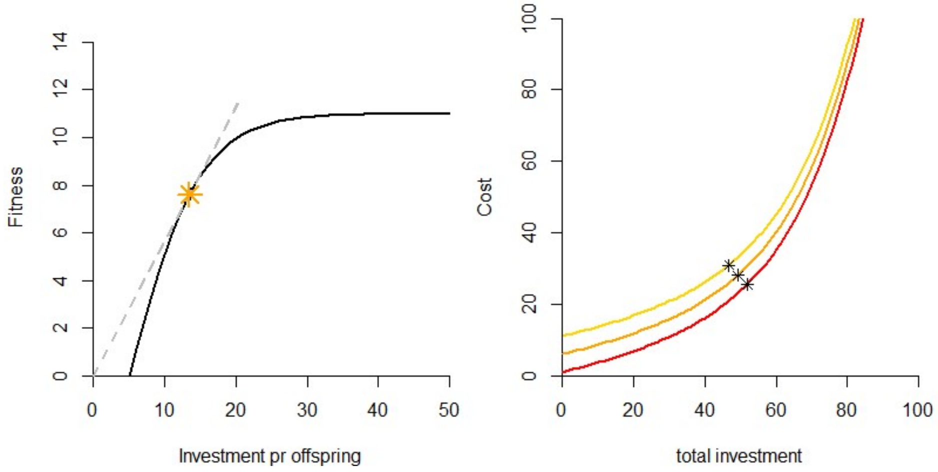

Figure S21. Scenario 2A. A) Offspring fitness function for all different quality males dependent on maternal investment per offspring. Tangent from origin to the fitness function is drawn in a grey dotted line. The star indicate the average of the optimal investment for the 80 highest maternal states for all different male qualities from the stochastic dynamic optimization model. B) Maternal cost functions for the three different quality males, given total reproductive investment (yellow: low quality, orange: medium quality, red: high quality). Stars indicate the cost for the optimal investment of females paired with the three different quality males from the stochastic dynamic model.

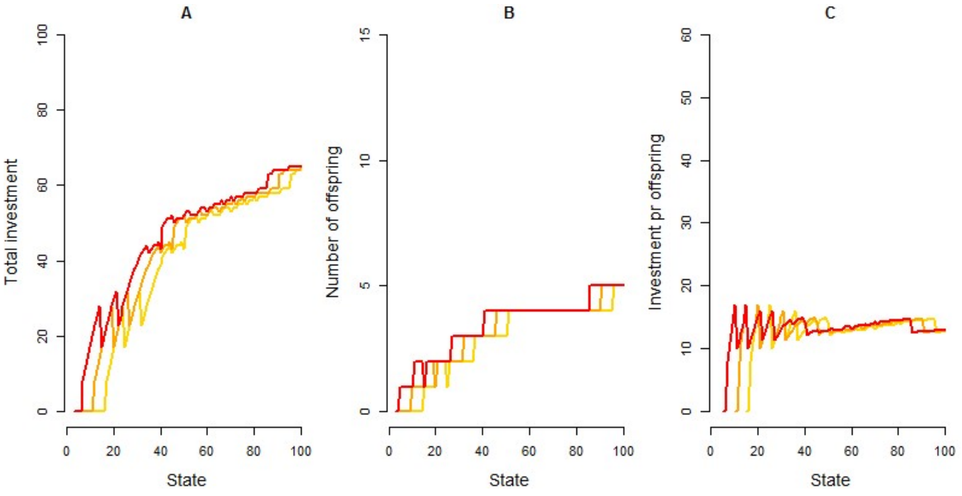

Figure S22. Plots showing state dependent results from the stochastic dynamic optimization model for scenario 2A. Each panel shows the different optimal investments along the axis of female energetic state (x-axis). Yellow lines correspond to low quality males, orange medium quality and red high quality males. A) Total investments given maternal energetic state. B) Number of offspring given maternal energetic state. C) Investment per offspring given maternal energetic state. Lines in A) are products of the lines in B) and C).

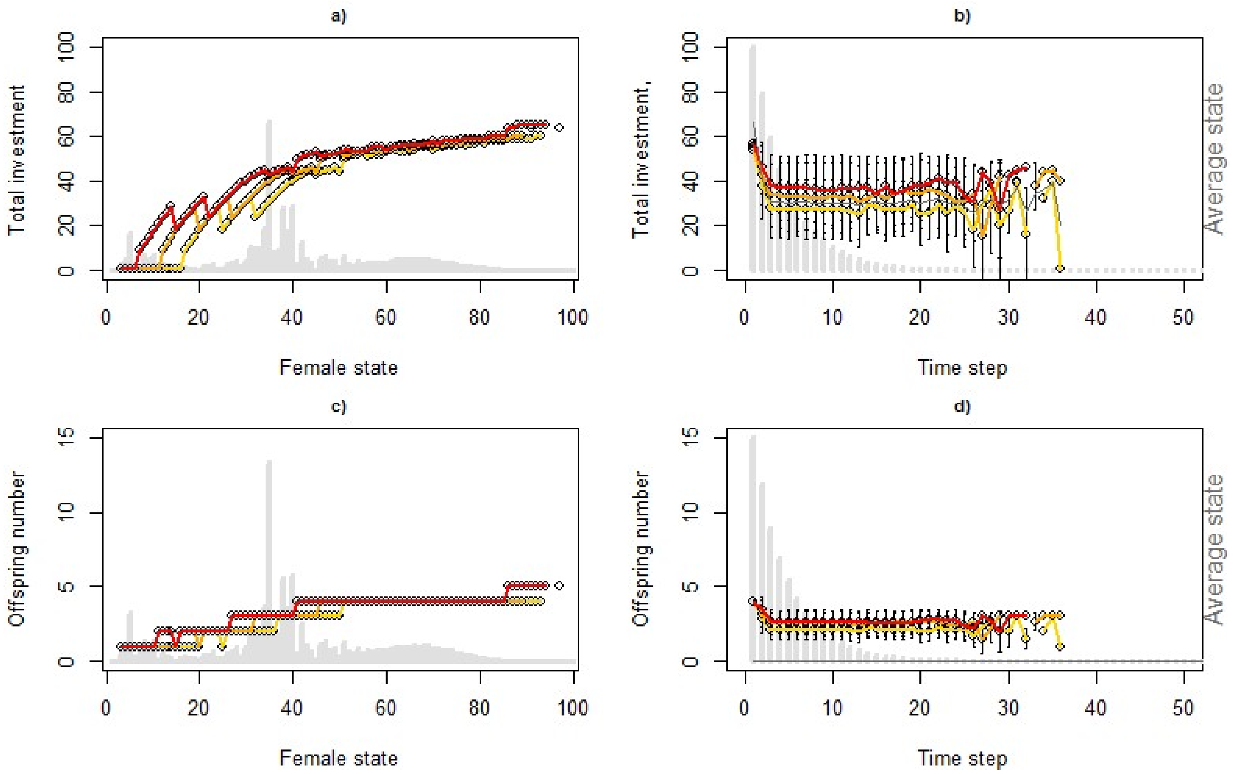

Figure S23. Results from simulation model for scenario 2A. In all panels red lines represent investments in offspring from high quality males, orange for medium quality males and yellow for low quality males. Grey bars in the background show distributions of females in different states (a and c) and over different time steps (b and d). Black error bars show standard deviations. a) Total investment in relation to maternal energetic state. b) Total investment over time. c) Number of offspring in relation to maternal energetic state. d) Number of offspring over time.

Scenario 2B: Male quality effect on cost function slope

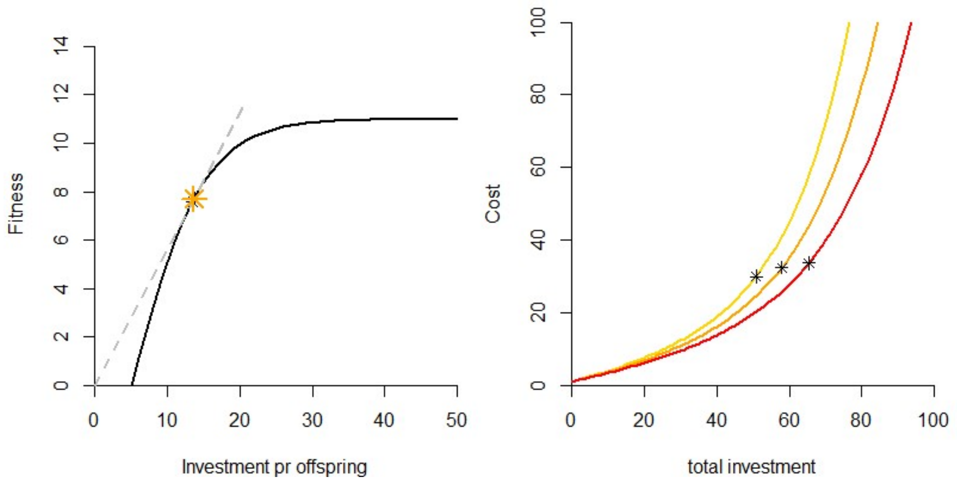

Figure S24. Scenario 2B. A) Offspring fitness function for all different quality males dependent on maternal investment per offspring. Tangent from origin to the fitness function is drawn in a grey dotted line. The star indicate the average of the optimal investment for the 80 highest maternal states for all different male qualities from the stochastic dynamic optimization model. B) Maternal cost functions for the three different quality males, given total reproductive investment (yellow: low quality, orange: medium quality, red: high quality). Stars indicate the cost for the optimal investment of females paired with the three different quality males from the stochastic dynamic model.

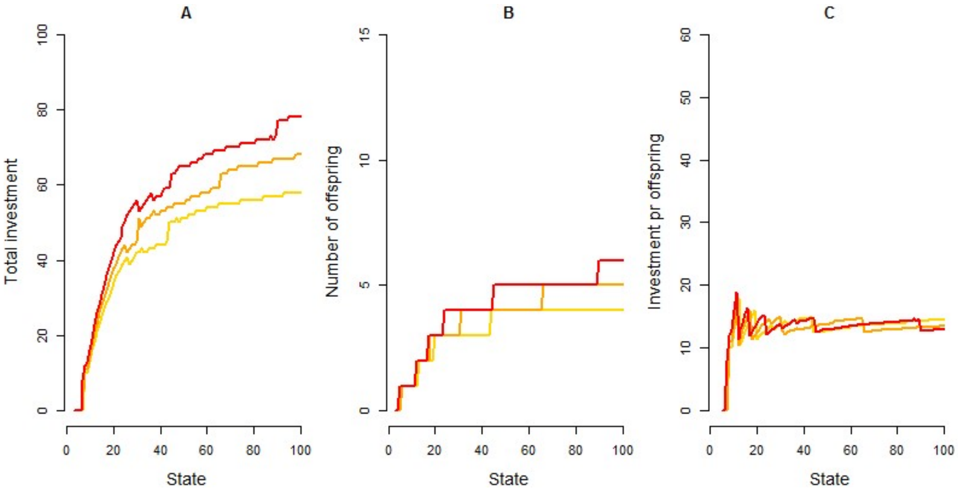

Figure S25. Plots showing state dependent results from the stochastic dynamic optimization model for scenario 2B. Each panel shows the different optimal investments along the axis of female energetic state (x-axis). Yellow lines correspond to low quality males, orange medium quality and red high quality males. A) Total investments given maternal energetic state. B) Number of offspring given maternal energetic state. C) Investment per offspring given maternal energetic state. Lines in A) are products of the lines in B) and C).

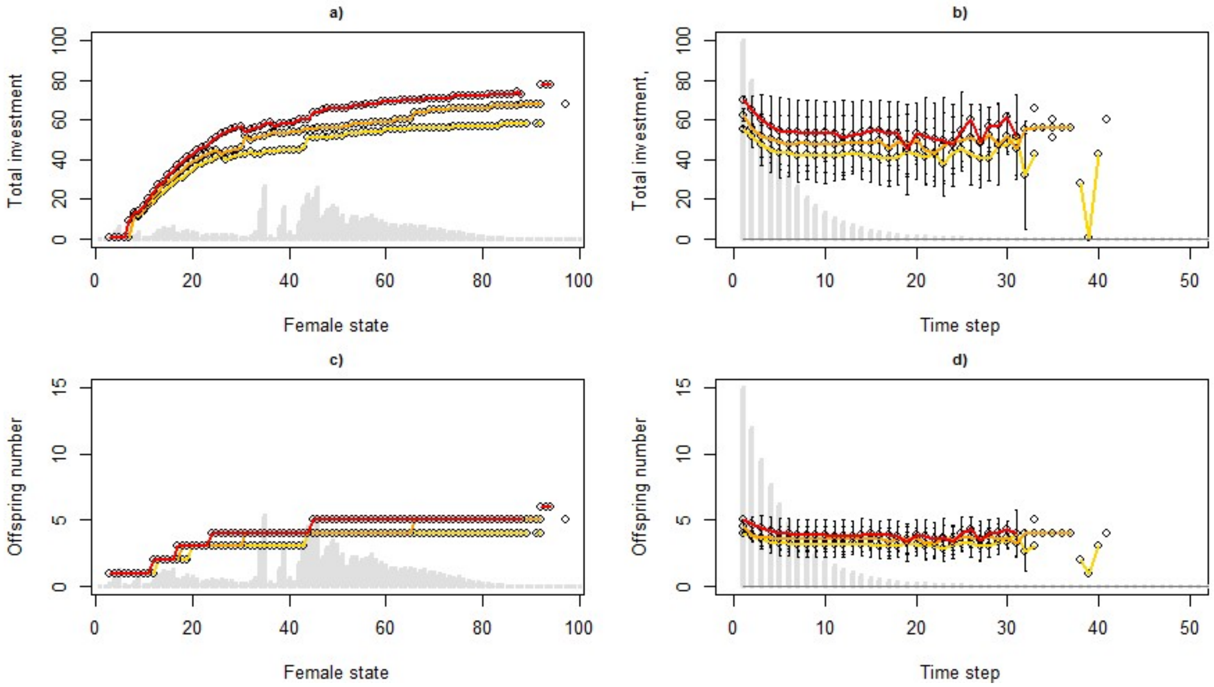

Figure S26. Results from simulation model for scenario 2B. In all panels red lines represent investments in offspring from high quality males, orange for medium quality males and yellow for low quality males. Grey bars in the background show distributions of females in different states (a and c) and over different time steps (b and d). Black error bars show standard deviations. a) Total investment in relation to maternal energetic state. b) Total investment over time. c) Number of offspring in relation to maternal energetic state. d) Number of offspring over time.

Scenario 2C: Male quality effects on position of cost function

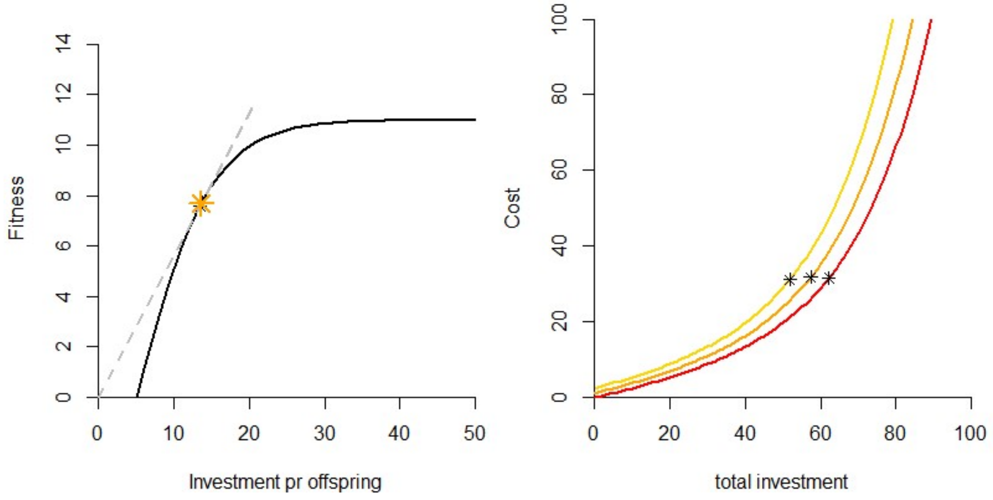

Figure S27. Scenario 2C. A) Offspring fitness function for all different quality males dependent on maternal investment per offspring. Tangent from origin to the fitness function is drawn in a grey dotted line. The star indicate the average of the optimal investment for the 80 highest maternal states for all different male qualities from the stochastic dynamic optimization model. B) Maternal cost functions for the three different quality males, given total reproductive investment (yellow: low quality, orange: medium quality, red: high quality). Stars indicate the cost for the optimal investment of females paired with the three different quality males from the stochastic dynamic model.

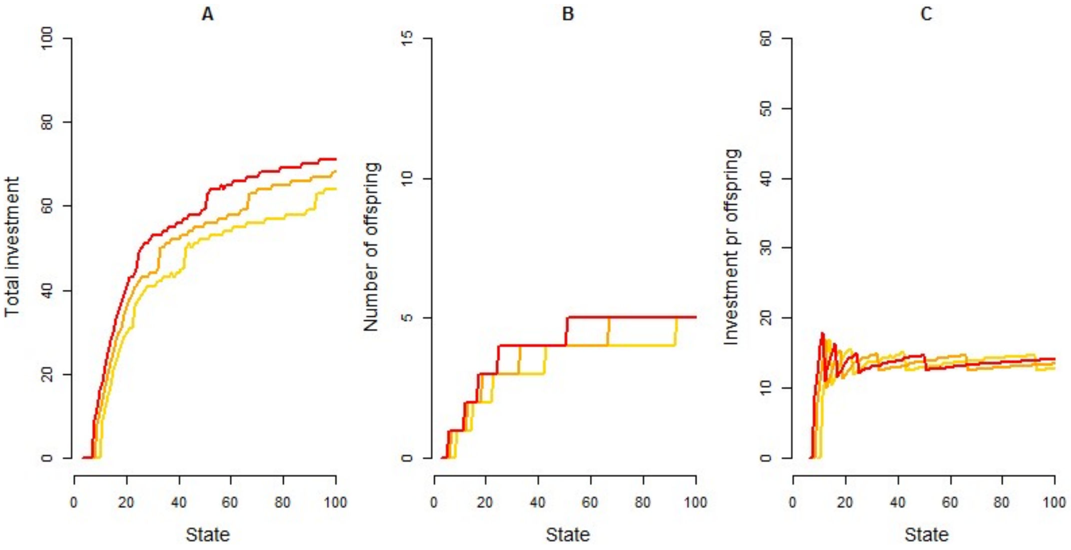

Figure S28. Plots showing state dependent results from stochastic dynamic optimization model for scenario 2C. Each panel shows the different optimal investments along the axis of female energetic state (x-axis). Yellow lines correspond to low quality males, orange medium quality and red high quality males. A) Total investments given maternal energetic state. B) Number of offspring given maternal energetic state. C) Investment per offspring given maternal energetic state. Lines in A) are products of the lines in B) and C).

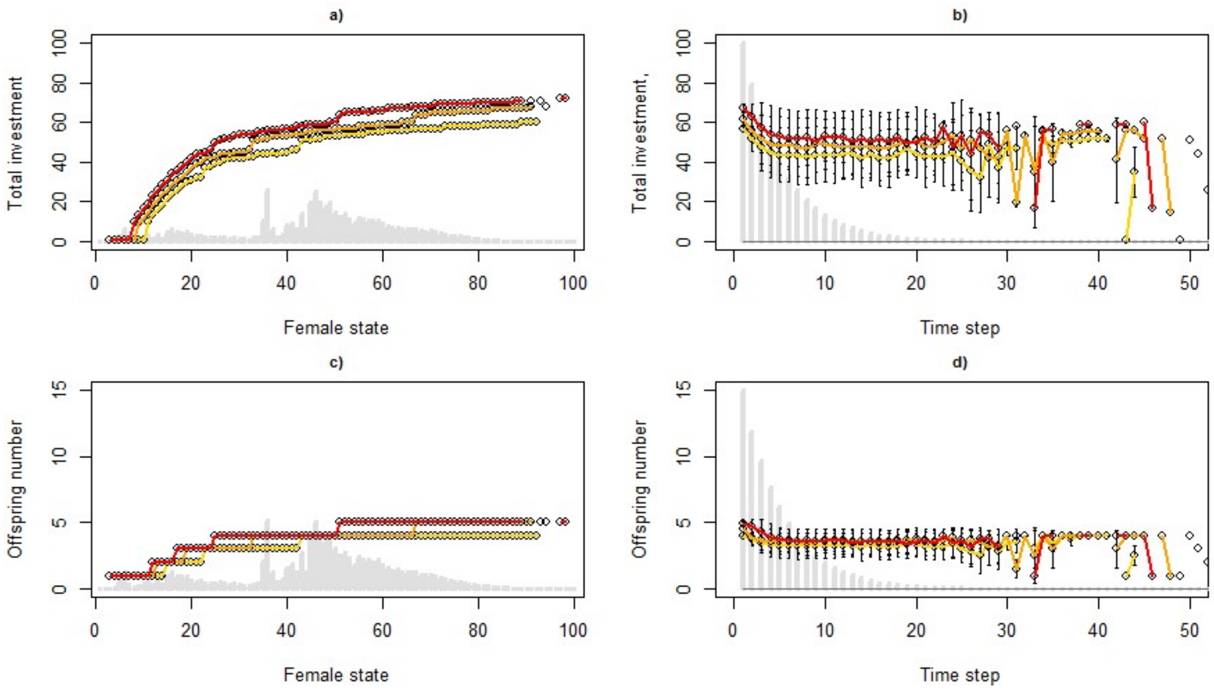

Figure S29. Results from simulation model for scenario 2C. In all panels red lines represent investments in offspring from high quality males, orange for medium quality males and yellow for low quality males. Grey bars in the background show distributions of females in different states (a and c) and over different time steps (b and d). Black error bars show standard deviations. a) Total investment in relation to maternal energetic state. b) Total investment over time. c) Number of offspring in relation to maternal energetic state. d) Number of offspring over time.

Scenario 2D: Male quality combined effects on cost function elevation and slope

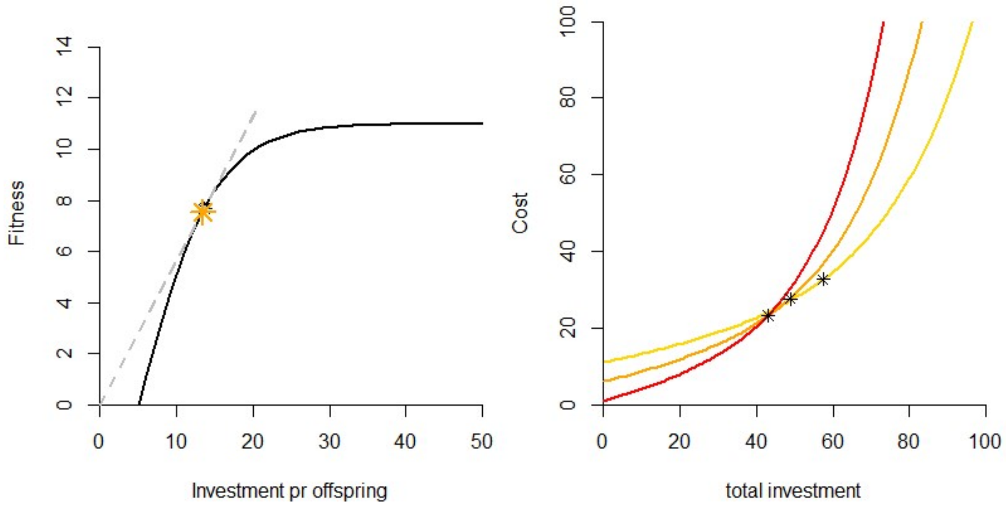

Figure S30. Scenario 2D. A) Offspring fitness function for all different quality males dependent on maternal investment per offspring. Tangent from origin to the fitness function is drawn in a grey dotted line. The star indicate the average of the optimal investment for the 80 highest maternal states for all different male qualities from the stochastic dynamic optimization model. B) Maternal cost functions for the three different quality males, given total reproductive investment (yellow: low quality, orange: medium quality, red: high quality). Stars indicate the cost for the optimal investment of females paired with the three different quality males from the stochastic dynamic model.

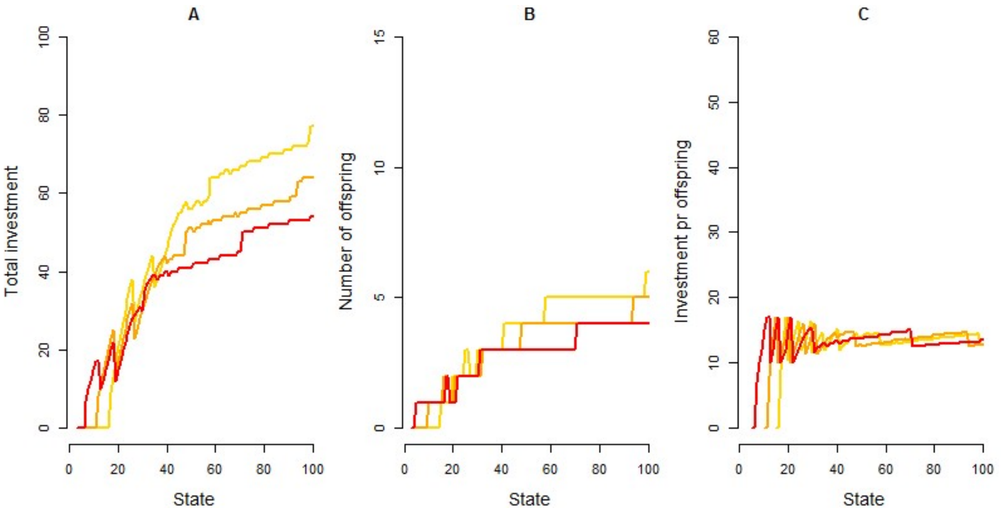

Figure S31. Plots showing state dependent results from stochastic dynamic optimization model for scenario 2D. Each panel shows the different optimal investments along the axis of female energetic state (x-axis). Yellow lines correspond to low quality males, orange medium quality and red high quality males. A) Total investments given maternal energetic state. B) Number of offspring given maternal energetic state. C) Investment per offspring given maternal energetic state. Lines in A) are products of the lines in B) and C).

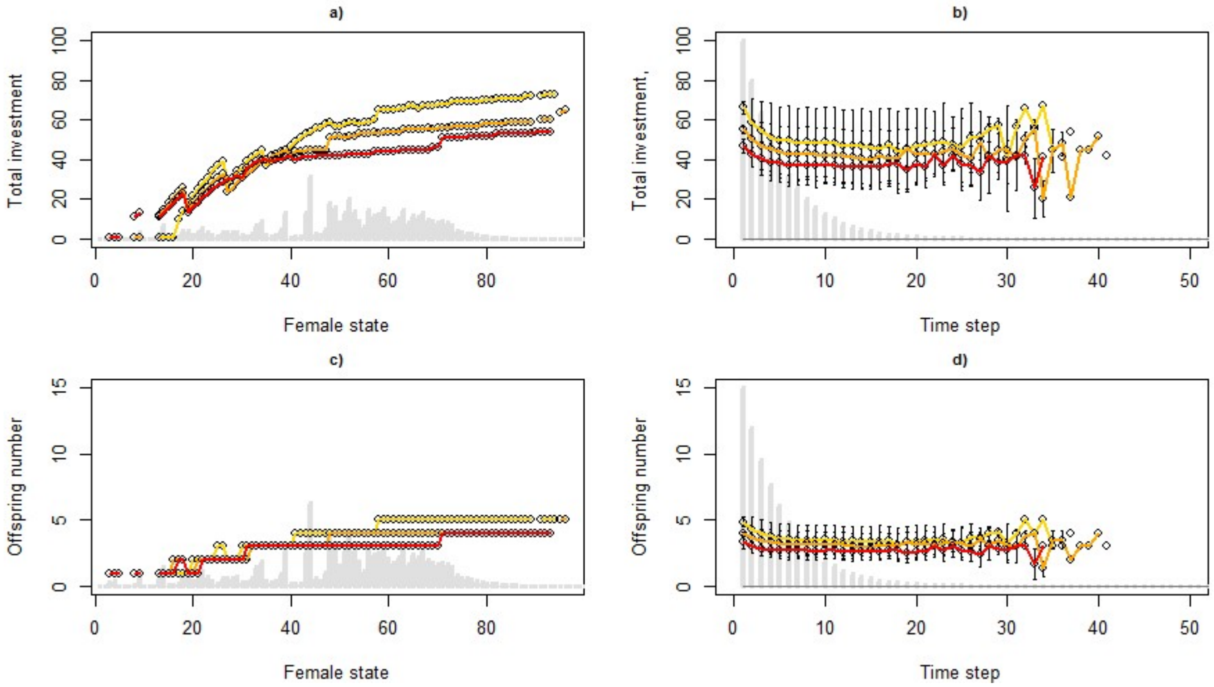

Figure S32. Results from simulation model for scenario 2D. In all panels red lines represent investments in offspring from high quality males, orange for medium quality males and yellow for low quality males. Grey bars in the background show distributions of females in different states (a and c) and over different time steps (b and d) Black error bars show standard deviations. a) Total investment in relation to maternal energetic state. b) Total investment over time. c) Number of offspring in relation to maternal energetic state. d) Number of offspring over time.

Scenario 3A*:* Male quality effects on both offspring fitness and female cost function

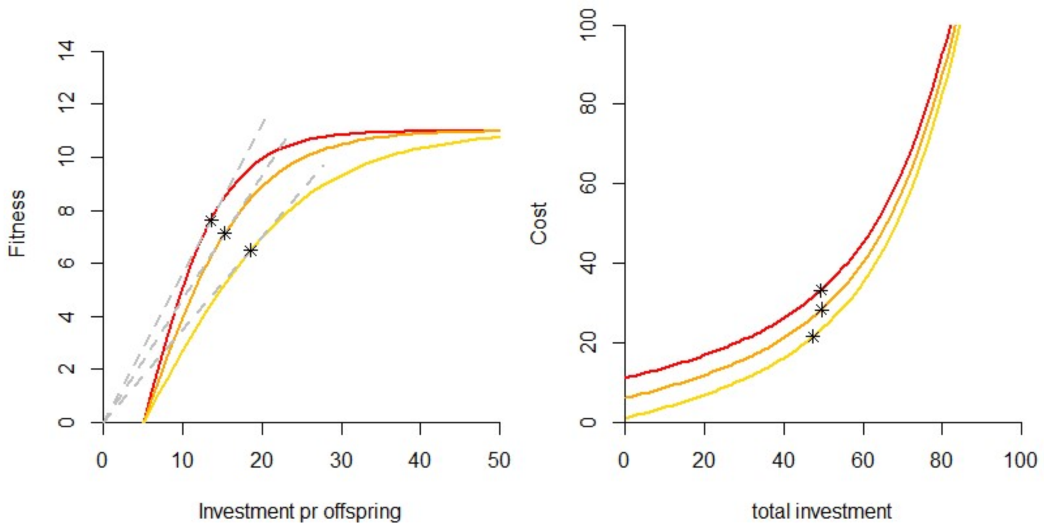

Figure S33. Scenario 3A. A) Offspring fitness functions for different quality males dependent on maternal investment per offspring. Tangents from origin to each of the fitness functions are drawn in grey dotted lines. The stars indicate the average of the optimal investment for the 80 highest maternal states for the three different male qualities from the stochastic dynamic optimization model. B) Maternal cost functions for the three different quality males, given total reproductive investment (yellow: low quality, orange: medium quality, red: high quality). Stars indicate the cost for the optimal investment of females paired with the three different quality males from the stochastic dynamic model.

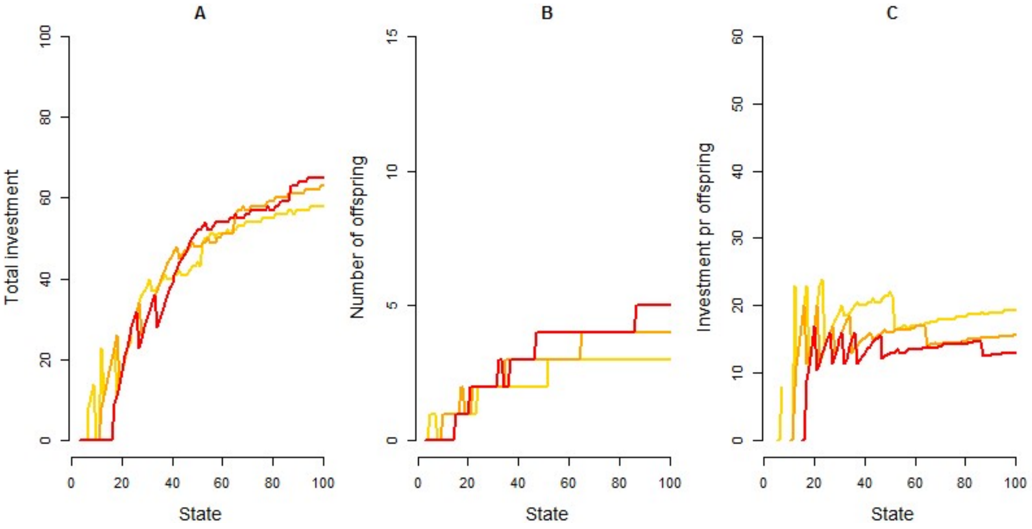

Figure S34. Plots showing state dependent results from the stochastic dynamic optimization model for scenario 3A. Each panel shows the different optimal investments along the axis of female energetic state (x-axis). Yellow lines correspond to low quality males, orange medium quality and red high quality males. A) Total investments given maternal energetic state. B) Number of offspring given maternal energetic state. C) Investment per offspring given maternal energetic state. Lines in A) are products of the lines in B) and C).

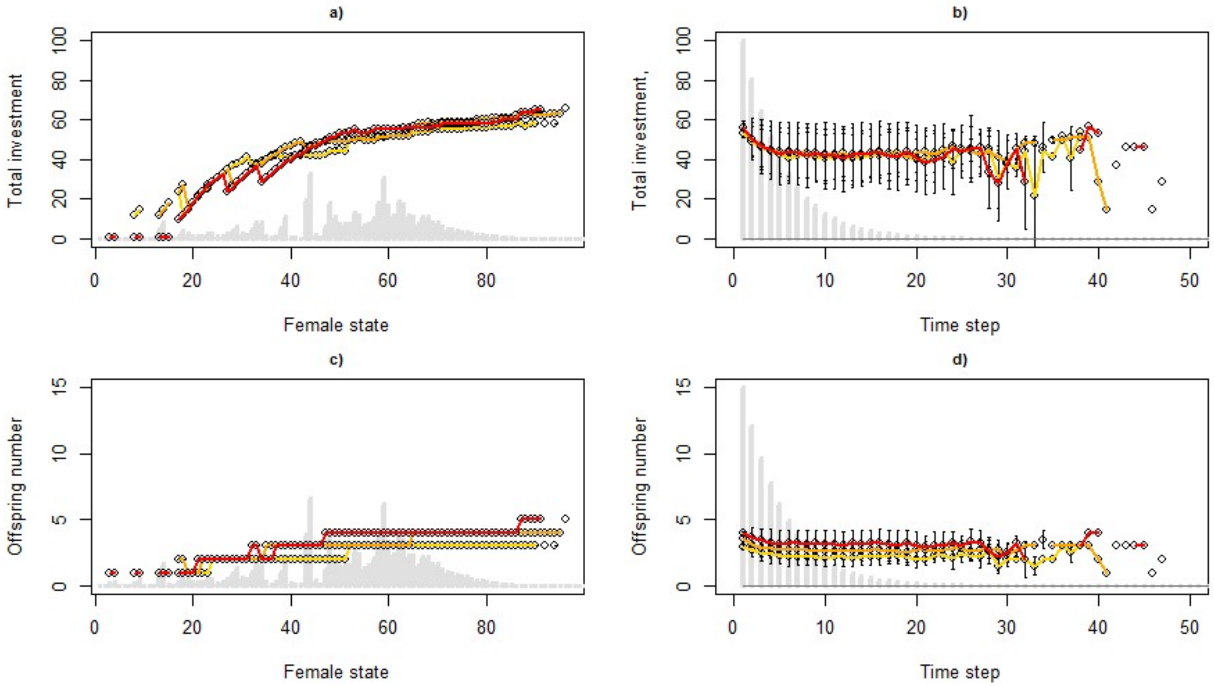

Figure S35. Results from simulation model for scenario 3A. In all panels red lines represent investments in offspring from high quality males, orange for medium quality males and yellow for low quality males. Grey bars in the background show distributions of females in different states (a and c) and over different time steps (b and d). Black error bars show standard deviations. a) Total investment in relation

to maternal energetic state. b) Total investment over time. c) Number of offspring in relation to maternal energetic state. d) Number of offspring over time
